# FgPfn participates in vegetative growth, sexual reproduction, pathogenicity, and fungicides sensitivity via affecting both microtubules and actin in the filamentous fungus *Fusarium graminearum*

**DOI:** 10.1371/journal.ppat.1012215

**Published:** 2024-05-03

**Authors:** Zhili Yuan, Pengfei Li, Xin Yang, Xiaowei Cai, Luoyu Wu, Feifei Zhao, Weidong Wen, Mingguo Zhou, Yiping Hou

**Affiliations:** College of Plant Protection, Nanjing Agricultural University, Nanjing, Jiangsu, China; Purdue University, UNITED STATES

## Abstract

*Fusarium* head blight (FHB), caused by *Fusarium graminearum* species complexes (FGSG), is an epidemic disease in wheat and poses a serious threat to wheat production and security worldwide. Profilins are a class of actin-binding proteins that participate in actin depolymerization. However, the roles of profilins in plant fungal pathogens remain largely unexplored. Here, we identified FgPfn, a homolog to profilins in *F*. *graminearum*, and the deletion of *FgPfn* resulted in severe defects in mycelial growth, conidia production, and pathogenicity, accompanied by marked disruptions in toxisomes formation and deoxynivalenol (DON) transport, while sexual development was aborted. Additionally, FgPfn interacted with Fgα_1_ and Fgβ_2_, the significant components of microtubules. The organization of microtubules in the Δ*FgPfn* was strongly inhibited under the treatment of 0.4 μg/mL carbendazim, a well-known group of tubulin interferers, resulting in increased sensitivity to carbendazim. Moreover, FgPfn interacted with both myosin-5 (FgMyo5) and actin (FgAct), the targets of the fungicide phenamacril, and these interactions were reduced after phenamacril treatment. The deletion of *FgPfn* disrupted the normal organization of FgMyo5 and FgAct cytoskeleton, weakened the interaction between FgMyo5 and FgAct, and resulting in increased sensitivity to phenamacril. The core region of the interaction between FgPfn and FgAct was investigated, revealing that the integrity of both proteins was necessary for their interaction. Furthermore, mutations in R72, R77, R86, G91, I101, A112, G113, and D124 caused the non-interaction between FgPfn and FgAct. The R86K, I101E, and D124E mutants in FgPfn resulted in severe defects in actin organization, development, and pathogenicity. Taken together, this study revealed the role of FgPfn-dependent cytoskeleton in development, DON production and transport, fungicides sensitivity in *F*. *graminearum*.

## Author summary

Profilins, a class of cytoskeleton regulatory proteins, participate in actin depolymerization. Recent findings have also discovered that profilins could bind to microtubules in *vitro* and promote microtubule growth. However, their roles in plant fungal pathogens remain largely unknown. Here, we showed that the profilins homolog FgPfn was critical for the vegetative growth, sexual reproduction and pathogenicity in *Fusarium graminearum*. We found that FgPfn interacted with microtubules and participated in microtubule organization, and the microtubule organization was strongly inhibited by carbendazim in the absence of FgPfn. Our results indicated that FgPfn regulated the actin cytoskeleton by participating in the organization and interaction of myosin-5 (FgMyo5) and actin (FgAct). Moreover, we demonstrated that several amino acid mutations in FgPfn disrupted FgPfn-FgAct interaction, resulting in severe defects in actin organization, vegetative growth, and pathogenicity. Our results provided novel insights into the role of profilins in microtubule and actin organization. These conservative amino acids of FgPfn-FgAct interaction provide a potential molecular target for designing and developing novel fungicides.

## Introduction

The cytoskeleton is a highly dynamic structure, so the rapid and correct assembly of actin is essential for completing various cellular life activities [1,2]. In filamentous fungi, the actin cytoskeleton is intertwined to form three structures with different functions: Actin patches are mainly responsible for endocytosis, polar growth, and apical secretion [3–5]; actin cables are responsible for vesicle transport [6]; and actin rings regulate septum formation [6,7]. The actin patches at the tips of pathogenic filamentous fungal hyphae completed vigorous exocytosis activities [4]. There are many actin-interacting or related proteins involved in regulating actin dynamics, including actin nucleating factor Arp2/3 complex [8,9] and formin [10], actin capping proteins (CAPs) [11,12], actin depolymerizing factor (ADF)/cofilin family [13] and profilins [14].

Microtubules are a complex and highly dynamic cytoskeleton. They are usually hollow cylindrical structures composed of 13 parallel microtubule protofilaments [15]. The microtubules play a crucial role in the mitosis process of eukaryotes. It is also a track for intracellular transportation and can also participate in maintaining cell morphology, cell differentiation, cell motility and other life activities [16]. Microtubule is nucleated from the γ-tubulin ring complex and then assembled into hollow cylindrical polymers with α/β-tubulin heterodimers [17]. Although initially identified as an actin-sequestering protein, increasing evidence suggests that profilins bind to specific polyproline-rich regions in other actin-regulatory proteins [18], such as formins and Srv2 [19,20]. In recent years, profilin has been shown to contain specific residues that enable its direct interaction with microtubules [21]. These findings will increase our understanding of the regulatory mechanisms between actin and the microtubule cytoskeleton.

Profilin is a class of abundant and conserved proteins in eukaryotic cells and is a critical regulator of the actin depolymerization balance. Profilins mainly have three binding sites: actin-binding site, polyproline-binding site (PLP), and phosphatidylinositol-binding site (PtdIns (4,5) P_2_ or PIP_2_). The actin-binding site allows profilins to bind to ATP-G-actin, inhibits actin spontaneous nucleation, aids in the polymerization of ATP-G-actin to the positive end of F-actin, and also facilitates the conversion of ADP-G-actin to ATP-G-actin [22–24]. The PLP-binding site enables it to bind to specific polyproline-rich regions in other actin-regulatory proteins, such as formins, Srv2, and WASP [19,20,25,26]. The interaction of profilin with PLP-containing proteins is crucial for the spatial-temporal regulation of actin polymerization. In yeast, the interaction between profilins and formins is crucial in delivering actin monomers to accelerate barbed end elongation [27–29]. The latest research shows that profilins indirectly interact with microtubules through the formin-profilin complex to promote the coordination of actin and tubulin [30], or directly interact with microtubules through specific amino acid residues to promote microtubule elongation [21]. It was also found that actin and microtubule competitively bind profilins, which helps us understand the cooperative mechanism between the actin and microtubule cytoskeleton. Profilins contain two PIP_2_-binding sites that link profilins to the plasma membrane [31,32]. Notably, the binding of profilins to PIP_2_ and actin compete with each other, resulting in local changes in the plasma membrane composition that affect the regulation of the actin backbone by profilins [31,33].

*Fusarium* head blight (FHB) is an epidemic disease that reduces wheat yield quality. The secondary metabolites produced during the occurrence of FHB lead to harmful mycotoxin contamination in affected grains, such as deoxynivalenol (DON), seriously endangering food security and human and animal health [34]. Currently, the primary method of controlling FHB is the use of fungicides. The fungicide phenamacril is highly specific to *Fusarium* and has a well-control effect on FHB. Preliminary studies have shown that phenamacril acts on I-type myosin FgMyo5, inhibiting the ATPase activity of FgMyo5 and blocking the binding of FgMyo5 to FgAct [35–37]. The FgMyo5-FgAct cytoskeleton plays an essential role in DON biosynthesis, and inactivation of FgMyo5 by the fungicide phenamacril or disruption of F-actin formation with latrunculin-A significantly inhibited toxisomes formation and DON biosynthesis [38].

There has been interest in profilins for a long time [14,21,30–33]. However, the studies of profilins in plant fungal pathogens are lacking. In this study, we aimed to unravel the roles of profilins homolog FgPfn in the development and pathogenesis of the *Fusarium* head blight fungus *Fusarium graminearum*. Our results showed that FgPfn was essential for the development and pathogenicity of *F*. *graminearum*. Furthermore, FgPfn participated in microtubule organization by interacting with microtubules in *vivo* and regulated the organization of FgMyo5-FgAct cytoskeleton by interacting with FgMyo5 and FgAct. Moreover, we investigated the core amino acids involved in the interaction between FgPfn and FgAct, revealing that R72, R77, R86, G91, I101, A112, G113 and D124 in FgPfn were crucial for the FgPfn-FgAct interaction. At the same time, R86K, I101E and D124E mutations in FgPfn also caused severe defects in actin organization, development and pathogenicity. Therefore, we described the role of FgPfn in the regulation of microtubule cytoskeleton and FgMyo5-FgAct cytoskeleton, which was essential for the vegetative growth, sexual reproduction, DON production, and pathogenicity in *F*. *graminearum*.

## Results

### Identification of FgPfn as a cytoskeletal-protein binding protein

**Fig 1:**
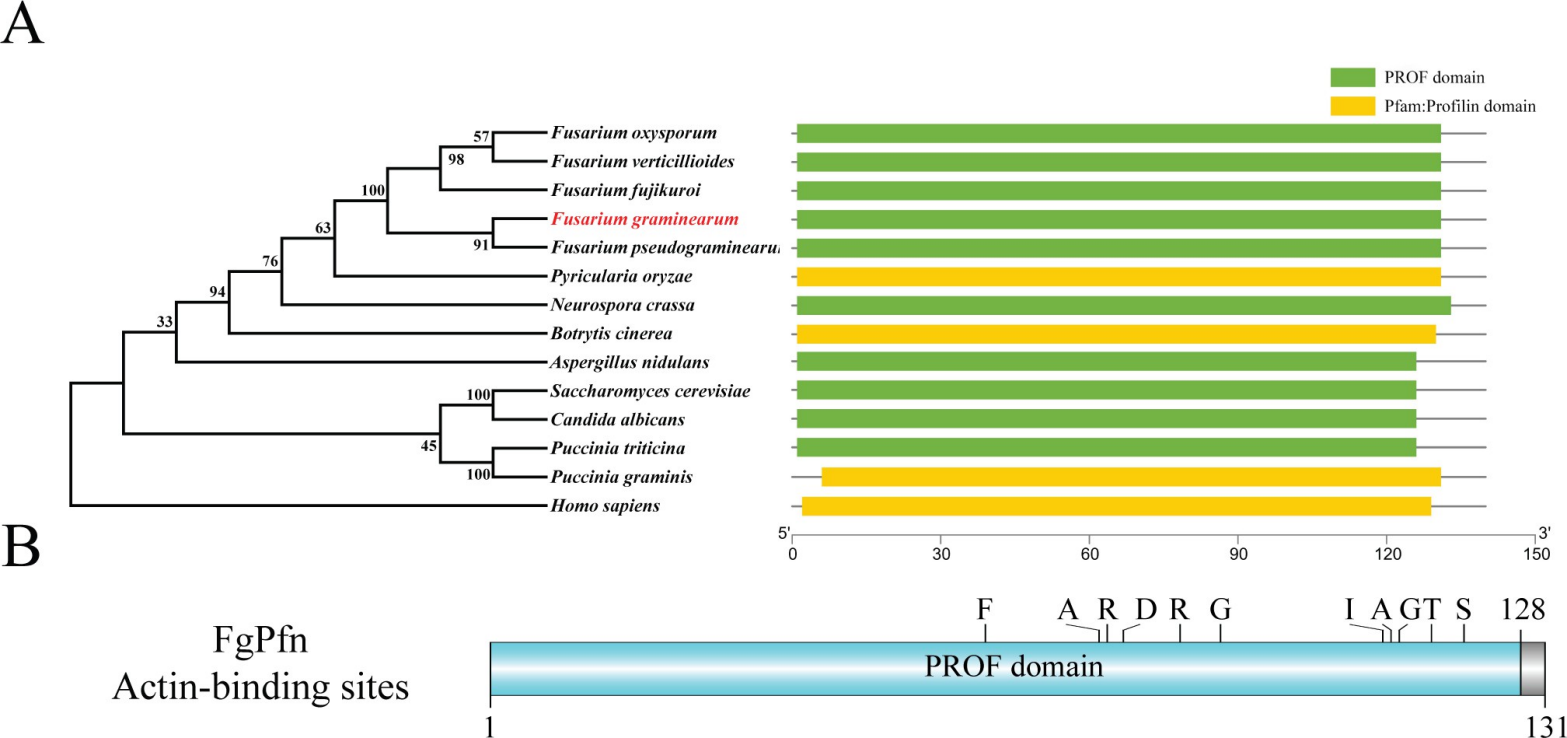
Identification of the FgPfn in *Fusarium graminearum*. (A) Phylogenetic and domain analysis of the profilins. Maximum Likelihood (ML) method, J Le and Gascuel (LG) model, and 1000 replicates were used to construct the phylogenetic tree by MEGA7. The domains were drawn with the Batch SMART in TBtools. (B) Domain and interaction sites analysis. With the CDD database online service function, the domains and actin-binding sites of FgPfn were drawn by DOG 2.0. The actin-binding sites include 11 amino acid residues in F62/A76/R77/D79/R86/G91/I111/A112/G113/T117/S121.

We identified the homologous protein FgPfn (FGSG_06392) of profilins from the IP-MS database of FgMyo5-GFP [39]. By searching the FungiDB database (an integrated functional genomics database for fungi, https://fungidb.org/fungidb/), FgPfn was predicted to encode a 131-amino acids protein that shares 46.21% identity with *S*. *cerevisiae* and shares 34.48% identity with *H*. *sapiens*. We retrieved profilins in other species from National Center for Biotechnology Information (NCBI), and the phylogenetic tree with domains of these sequences was analyzed by TBtools (Fig 1A). Although profilins were conserved in eukaryotes, the phylogenetic tree showed that FgPfn was not highly homologous with other organisms. All profilins contain a PROF domain, a very conserved domain of the profilins family. Further analysis showed that FgPfn contains actin-binding sites, PLP (polyproline)-binding sites, and PIP_2_ (phosphatidylinositol)-binding sites by the CDD database (http://www.ncbi.nlm.nih.gov/Structure/cdd/wrpsb.cgi/), of which 11 actin-binding sites were shown in Fig 1B.

### FgPfn is essential for vegetative growth

Targeted gene replacement was performed using the split-marker strategy to characterize FgPfn functionally. Two independent Δ*FgPfn* mutants, Δ*FgPfn-6* and Δ*FgPfn-8*, were obtained, and both displayed similar defects. To confirm that the changes observed in Δ*FgPfn* were caused by gene deletion, the Δ*FgPfn* mutant was complemented using a full-length *FgPfn* gene with a 3×Flag tag fusion vector. Complementation mutant Δ*FgPfn*-C-3×Flag was sequenced and further confirmed by Southern blot (S1 Fig).

**Fig 2:**
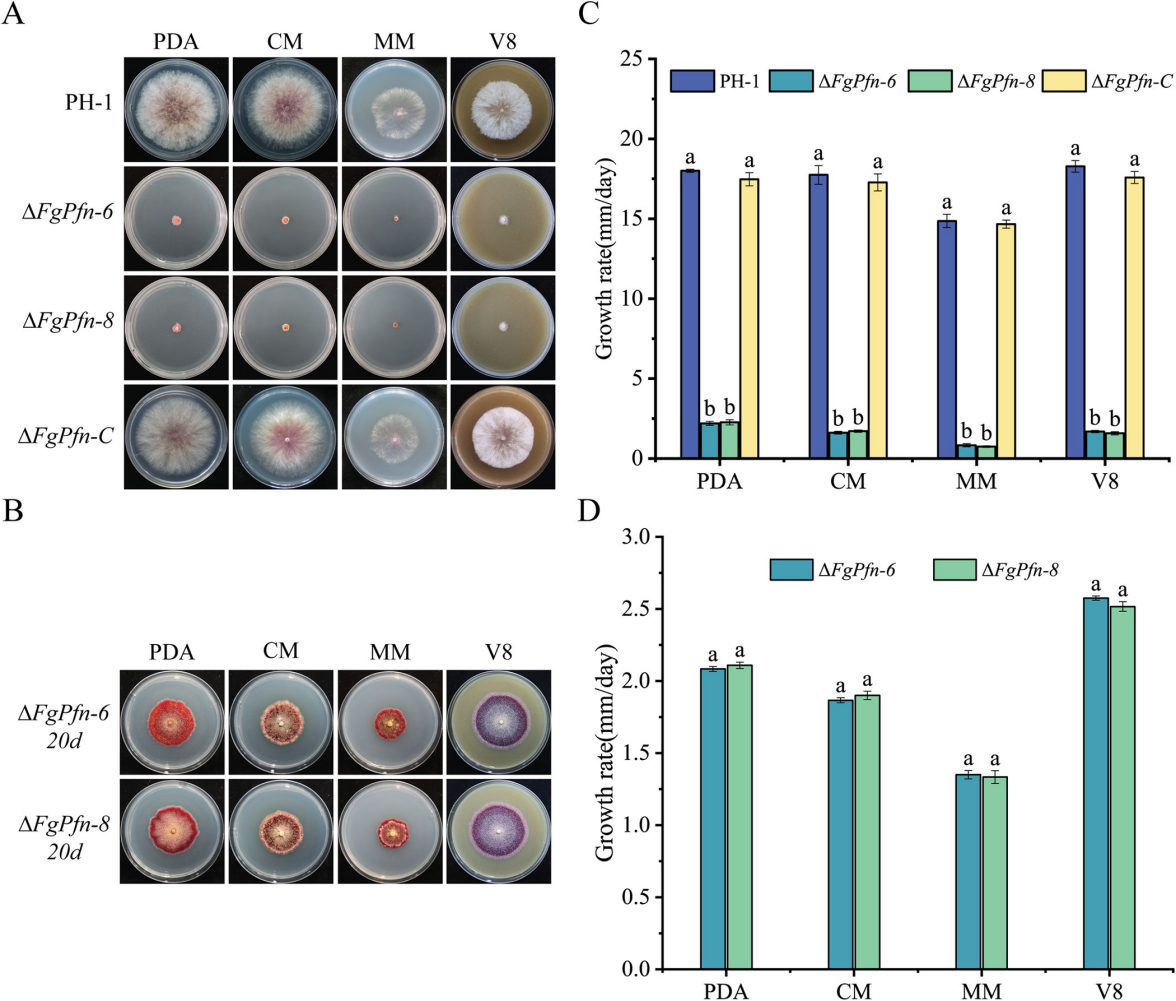
FgPfn is essential for vegetative growth. (A) Growth phenotype of Δ*FgPfn* mutants. The colony morphology was photographed after the 3d inoculation of each strain on PDA, CM, MM, and V8 medium. (B) The colony morphology was photographed after the 20d inoculation of mutants on PDA, CM, MM, and V8 medium. (C) Mycelial growth rate of each strain was measured after the 3d growth on PDA, CM, MM, and V8 medium. Bars with the same letter indicate no significant difference according to the least significant difference (LSD) test at p < 0.05. (D) Mycelial growth rate of each mutant was measured after the 20d growth on PDA, CM, MM, and V8 medium. Bars with the same letter indicate no significant difference according to the LSD test at p < 0.05.

To investigate the role of FgPfn in the vegetative growth of *F*. *graminearum*, we performed growth assays of mutants on different medium (Fig 2). Compared with PH-1, the growth rates of the Δ*FgPfn* deletion mutants were significantly reduced by about 90% on potato dextrose agar (PDA), complete medium (CM), minimum medium (MM), and V8 medium. After the Δ*FgPfn* deletion mutants were cultured for 20 d, the colony diameter could reach about 40 mm on PDA medium. The complementation mutant Δ*FgPfn*-C restored the growth defect caused by *FgPfn* deletion. The results indicated that FgPfn was involved in the vegetative development of *F*. *graminearum*.

### FgPfn is necessary for asexual and sexual development

**Fig 3:**
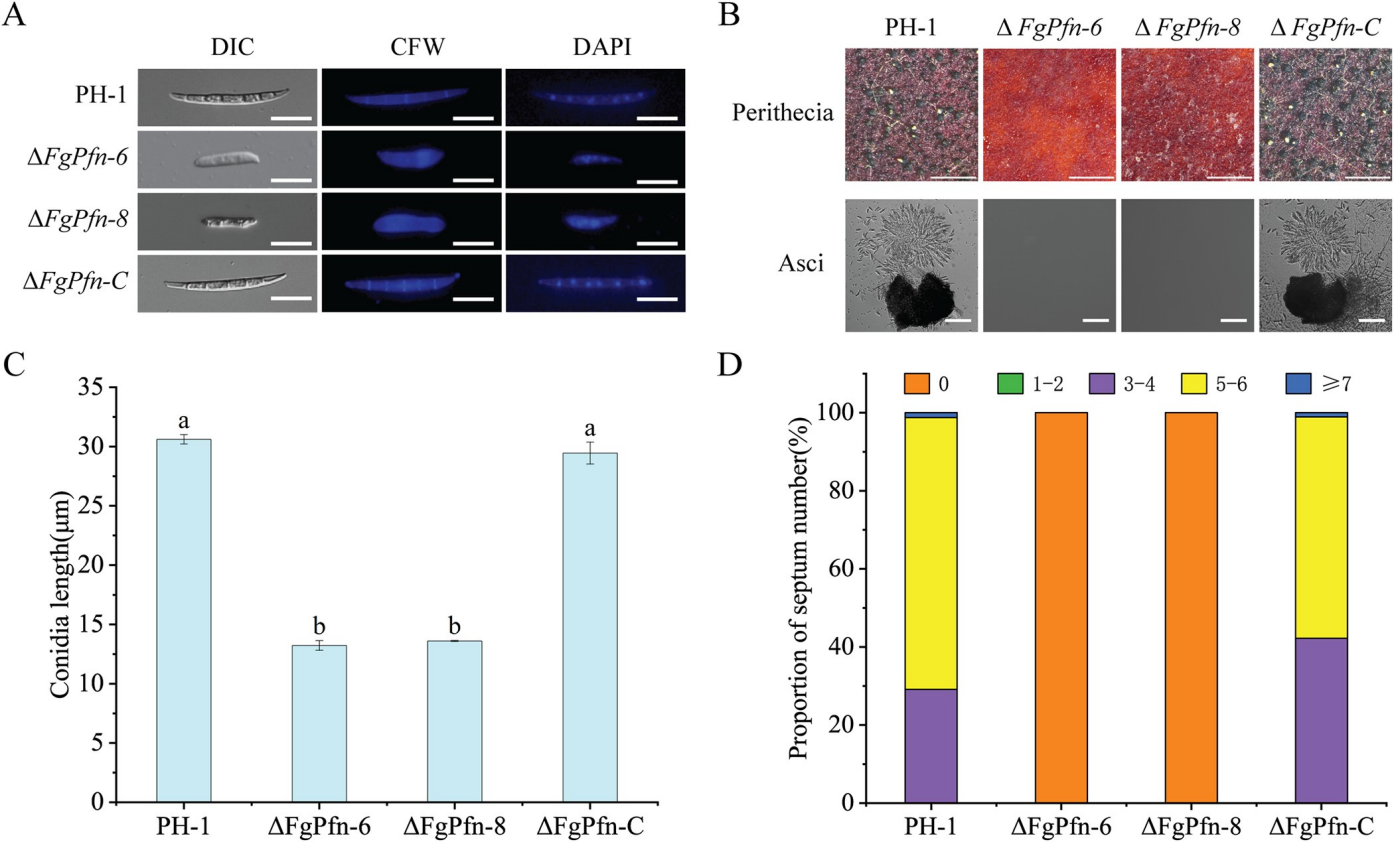
Deletion of *FgPfn* causes defects in asexual and sexual development. (A) Deletion of *FgPfn* results in conidial morphological defects. Conidia were examined by differential interference contrast (DIC) microscopy. The septum of conidia was stained with Calcofluor white (CFW) and photographed with an inverted fluorescent microscope at 20×. The nuclei of conidia were stained with 4’,6-DiAmidino-2-PhenylIndole (DAPI) for 30 min and photographed with an inverted fluorescent microscope at 20×. Bar = 10 μm. (B) Sexual development of Δ*FgPfn*. Fresh mycelia of each strain were inoculated on carrot agar medium (CA) plates and cultivated in the dark at 25°C, in which PH-1 and complementation strain were cultured for 6d, Δ*FgPfn* mutants were inoculated in advance and cultured for 15d. All strains were scraped aerial mycelium simultaneously and treated with 2.5% Tween-20. After being cultivated under fluorescent light for 7-14d, the perithecia were photographed using a stereo microscope. Bar = 1 000 μm. Then the perithecia were crushed on the slides to observe the asci. Bar = 10 μm. (C) The average conidia length of each strain was measured with 100 conidia, which was three repeated times. Bars with the same letter indicate no significant difference according to the LSD test at p < 0.05. (D) The number of conidial septa of each strain was counted after staining with CFW, and then the proportion of conidia with different septate numbers to the total number was calculated. Count 100 conidia for each strain and repeat three times. Bars with the same letter indicate no significant difference according to the LSD test at p < 0.05.

In order to determine the role of FgPfn in asexual development, the Δ*FgPfn* mutants were cultured in mung bean soup medium to produce conidia. Δ*FgPfn* mutants could still produce conidia. Nevertheless, compared with PH-1, there were severe defects in producing conidia with significantly reduced conidiation (Table 1) and conidia length (Fig 3A and 3C). Furthermore, conidia of the Δ*FgPfn* mutants did not form septa but had multiple nuclei (Fig 3A and 3D). However, the germination rate of Δ*FgPfn* deletion mutant was not affected (Table 1). Δ*FgPfn*-C complementation mutant restored normal conidial ability and conidial morphology. The results indicated that FgPfn was involved in the asexual development, in which deletion mutant resulted in a significant defect in the conidia production and morphology but not in the germination.

**Table 1:** Conidiation and conidia germination of Δ*FgPfn* mutants.

Strain	Conidiation (1×10^5^)	Conidia germination rate (4 h) (%)
PH-1	12.72±0.40a	88.88±6.73a
Δ*FgPfn-6*	0.33±0.06b	92.00±2.58a
Δ*FgPfn*-8	0.24±0.06b	92.25±1.75a
Δ*FgPfn-*C	12.32±0.38a	89.50±4.58a

The same letter indicates no significant difference according to the LSD test at p < 0.05.

To confirm whether FgPfn affected the sexual development of *F*. *graminearum*, we determined the sexual development ability of Δ*FgPfn*. The strains were inoculated into carrot medium plates and cultured under fluorescent lamps (PHILIPS, TLD 30W/54-765) for 7d (12h light, 12h dark). The Δ*FgPfn* deletion mutants could not produce perithecia on the 7th d, while the PH-1 and Δ*FgPfn*-C produced mature perithecia, asci, and ascospores (Fig 3B). Continue to culture the Δ*FgPfn* mutants for another 7d, and they still failed to produce perithecia. Therefore, FgPfn was very important for *F*. *graminearum* sexual development, in which the deletion mutant was unable to reproduce sexually.

### FgPfn affects the sensitivity to various stresses

**Fig 4:**
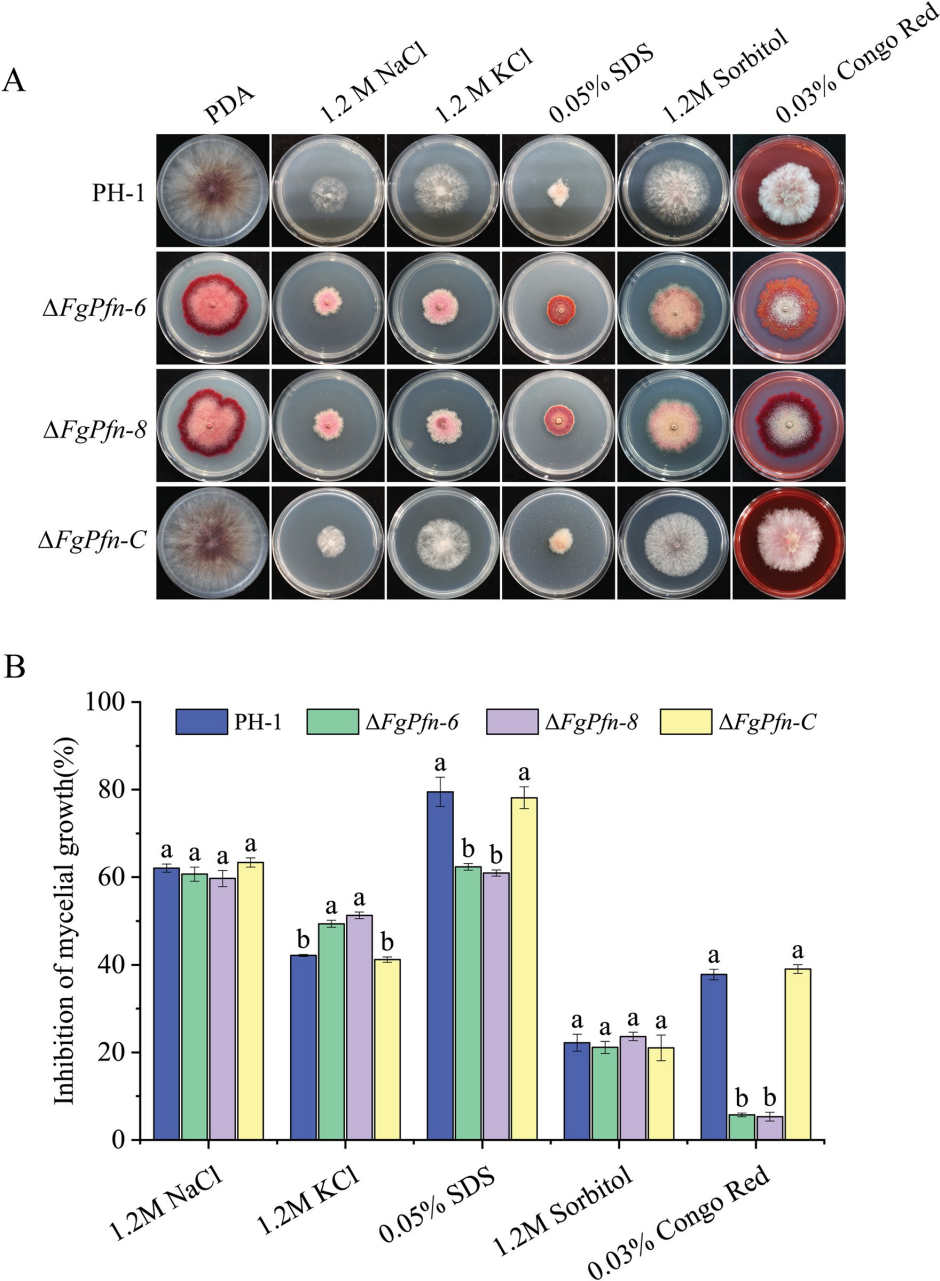
Sensitivity of Δ*FgPfn* mutants to different stress factors. (A) The colony morphology of Δ*FgPfn* mutants on different mediums. PH-1, Δ*FgPfn* mutants, and complementation strain were inoculated on PDA medium containing 1.2 M NaCl, 1.2 M KCl, 0.05% SDS, 1.2 M Sorbitol, and 0.03% Congo red at 25°C. The colony morphology of PH-1 and complementation strain was photographed after the 3d of growth, which was 20d in Δ*FgPfn*. (B) The inhibition rates of PH-1, Δ*FgPfn* mutants, and complementation strain with different stress factors. The hyphal growth inhibition rates of different stress factors on PH-1, Δ*FgPfn* mutants, and complementation strain were calculated with the colony diameters grown on PDA as control, where PH-1 and complementation mutant grew for 3d, and Δ*FgPfn* grew for 20d. Inhibition rates = (Average diameters of control group—Average diameters of treatment group)/Average diameters of control group × 100%. Bars with the same letter indicate no significant difference according to the LSD test at p < 0.05.

During the development and infection, *F*. *graminearum* faces different environmental stresses. So, we were interested in researching the sensitivities of the Δ*FgPfn* mutants to various stresses, including ionic stress, osmotic stress, cell membrane stress, and cell wall stresses generated by NaCl, KCl, Sorbitol, sodium dodecyl sulfate (SDS), and Congo Red (CR), respectively. As shown in Fig 4. Compared with PH-1, Δ*FgPfn* mutants exhibited increasing sensitivity to ionic stress of KCl, decreased sensitivity to cell membrane stress SDS and cell wall stress Congo red. At the same time, there was no significant difference in the sensitivity to ionic stress NaCl and osmotic stress Sorbitol. The complementation mutant Δ*FgPfn*-C exhibited similar sensitivities to PH-1, indicating that FgPfn was involved in the regulation of *F*. *graminearum* to various stresses.

### FgPfn plays a crucial role in pathogenicity and toxisome assembly

**Fig 5:**
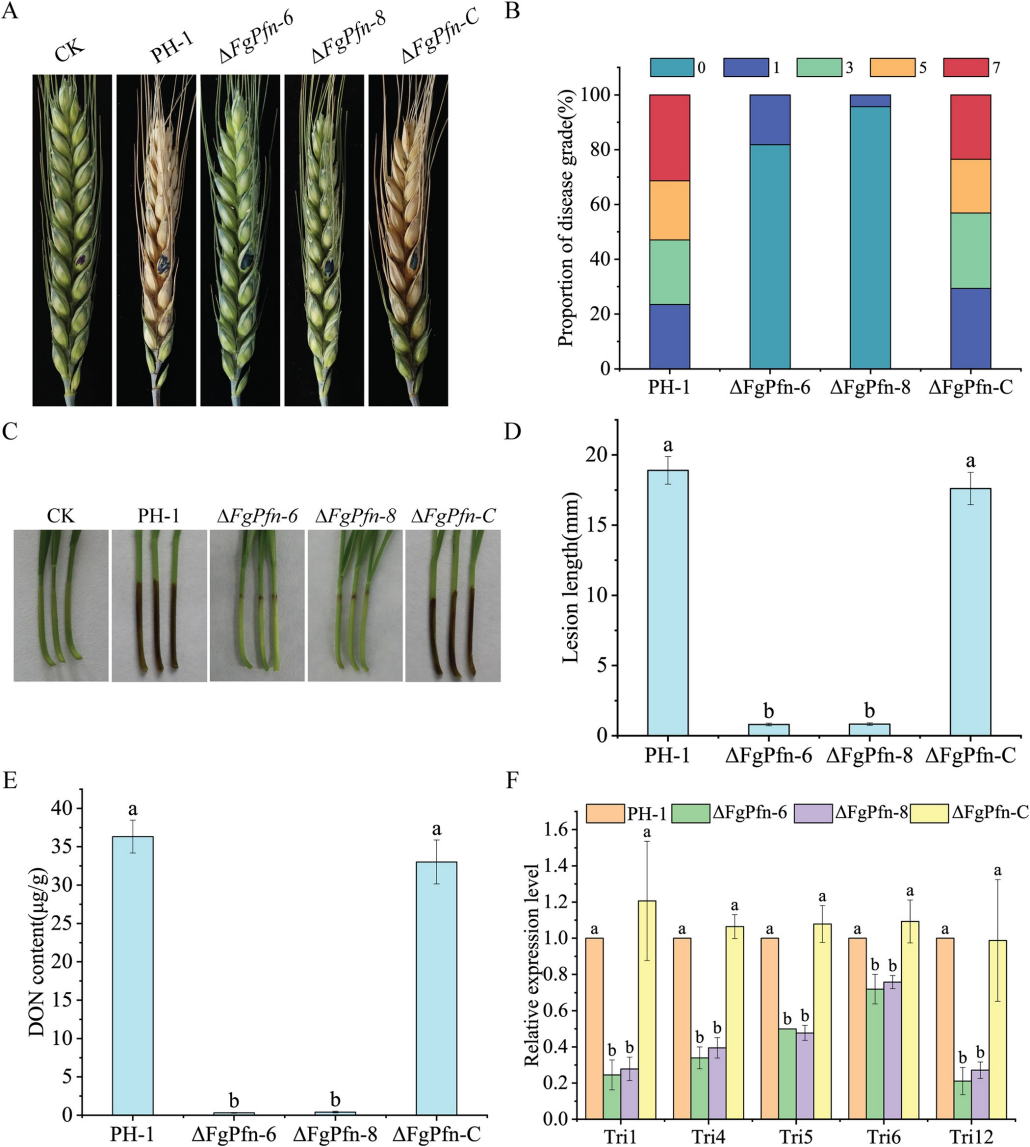
*FgPfn* deletion mutant attenuates pathogenicity and DON content. (A) Pathogenicity assays of Δ*FgPfn* mutants on wheat heads. At the blooming stage of wheat, 10 μL 1×10^6^ /mL conidia suspension was evaluated by point-inoculating in flowering wheat heads. After 14d, the incidence was investigated and the disease grade was counted. The wheat variety was Huaimai 20. (B) Proportion of disease grade, reflecting the severity of the disease: 0, disease free; 1, disease proportion is less than 25%; 3, disease proportion is between 25% and 50%; 5, disease proportion is between 50% and 75%; 7, disease proportion is more than75%. (C) Pathogenicity assays of Δ*FgPfn* mutants on wheat coleoptiles. Add 2 μL 1×10^6^ /mL conidia suspension were inoculated on the injured wheat coleoptile for 7d to observe the incidence and take photos. The wheat variety was Huaimai 33. (D) The average length of lesion on wheat coleoptile infected by each strain was measured after 7d post-inoculation. Bars with the same letter indicate no significant difference according to the LSD test at p < 0.05. (E) DON content assay of Δ*FgPfn* mutants. After the 7d of TBI culture, the DON content in the wild-type PH-1, Δ*FgPfn* mutants, and complementation strain were determined. Bars with the same letter indicate no significant difference according to the LSD test at p < 0.05. (F) Relative gene expression level of *TRI1*, *TRI4*, *TRI5*, *TRI6* and *TRI12* in the strains tested. After the 36h culture in TBI, mycelia of each strain were harvested for RNA extraction. The *GAPDH* was used as a reference gene. Bars with the same letter indicate no significant difference according to the LSD test at p < 0.05.

To assess the roles of FgPfn in the pathogenicity of *F*. *graminearum*, we carried out pathogenicity experiments on wheat heads and wheat coleoptiles. Flowering wheat heads were evaluated with disease grade two weeks after the point-inoculation of conidial suspensions from tested strains. Most wheat heads inoculated with the Δ*FgPfn* mutants showed no scab symptoms (Fig 5A and 5B). Only a few developed scab symptoms were restricted to the inoculated spikelets and failed to spread from the inoculated floret to the rachis. In contrast, severe and typical scab symptoms were caused by the wild-type PH-1 and complementation strain Δ*FgPfn*-C. Consistent with the results of wheat heads, Δ*FgPfn* could only lead to a mild disease at the inoculation site of the coleoptiles, which could not spread further compared with PH-1 and Δ*FgPfn*-C (Fig 5C and 5D). The results showed that FgPfn was essential for the pathogenicity of *F*. *graminearum*.

Deoxynivalenol (DON) is a key virulence factor in *F*. *graminearum* [38]. Since the pathogenicity of Δ*FgPfn* in wheat heads and coleoptiles was significantly reduced, we further determined the DON content in the DON-inducing trichothecene biosynthesis induction (TBI) medium at 28°C for 7d in the dark. The DON content of the Δ*FgPfn* mutants significantly declined compared to that of PH-1 and Δ*FgPfn*-C (Fig 5E), which was consistent with the reduced pathogenicity in Δ*FgPfn*. At the same time, the expression levels of five selected *TRIs* genes were assayed by qPCR after the 36h incubation in TBI medium. Compared with the wild-type strain, the expression levels of *TRIs* genes in the deletion mutants were significantly down-regulated (Fig 5F).

**Fig 6:**
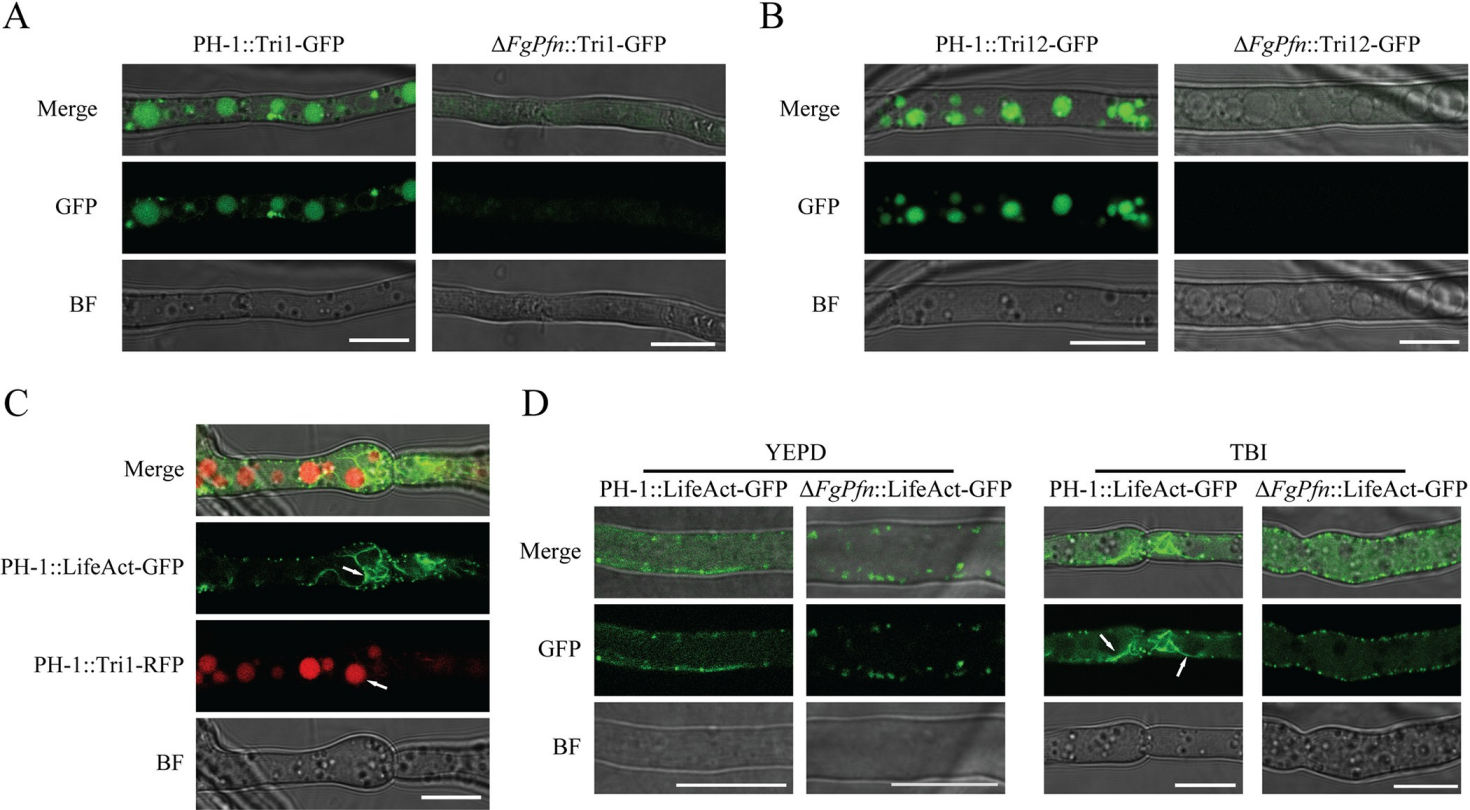
*FgPfn* deletion mutant disrupts toxisome formation and DON transfer. (A) Toxisome formation in Δ*FgPfn*. All strains were labeled with Tri1-GFP as a toxisome indicator and photographed under a confocal microscope after 36h of TBI culture. Bar = 10 μm. (B) The localization of Tri12-GFP in the PH-1 and Δ*FgPfn* mutant. All strains were labeled with Tri12-GFP and photographed under a confocal microscope after the 36h of TBI culture. Bar = 10 μm. (C) The association between toxisome and F-actin. Strain PH-1 co-expressing TRI1-RFP and LifeAct-GFP fusion proteins was photographed under a confocal microscope after 36h of TBI culture. Bar = 10 μm. (D) Actin organization in YEPD and TBI cultures. After the 36h of YEPD or TBI culture, the organization of actin marked by LifeAct-GFP in wild-type strain PH-1 and Δ*FgPfn* were photographed under a confocal microscope. Bar = 10 μm.

Toxisome is an essential compartment for the synthesis of DON, and Tri1-GFP can be used as an indicator for DON-toxisome formation [38]. To verify whether the reduction of DON in the Δ*FgPfn* mutant is related to the toxisome, we observed the localization of Tri1-GFP in the Δ*FgPfn* mutant. After the 36h incubation in TBI medium, the PH-1::Tri1-GFP strain formed typical spherical toxisomes, while Δ*FgPfn*::Tri1-GFP strain was unable to form toxisomes (Fig 6A). TRI12, a transporter protein localizing to the plasma membrane, vacuole, and small (∼1 μm) motile vesicles, interacts with toxisomes and may accumulate or transfer trichothecenes to the vacuole or export trichothecenes by exocytosis in *F*. *graminearum* under trichothecene induction conditions [34,40]. We found that the localization of Tri12-GFP in the PH-1 formed vacuole and small vesicles after the 36h incubation in TBI medium, while there was no fluorescence signal in the Δ*FgPfn*::Tri12-GFP strain (Fig 6B). The actin cytoskeleton is critical for toxisome formation and DON transport [38,40]. We constructed PH-1::LifeAct-GFP mutant and further expressed Tri1-RFP in the LifeAct-GFP genetic background to visualize actin and toxisomes under the conditions of trichothecene induction. It was found that actin accumulated around the toxisomes (Fig 6C). In contrast, the subcellular localization of actin was less in both PH-1 and Δ*FgPfn* under YEPD culture (Fig 6D left), indicating actin reorganization under TBI culture. However, the localization of actin in Δ*FgPfn*::LifeAct-GFP mutant failed to reorganize under TBI induction, similar to that in YEPD culture (Fig 6D right). The results showed that the deletion of *FgPfn* resulted in a failure of toxisome formation and DON transfer, which was essential for DON biosynthesis and pathogenicity in *F*. *graminearum*.

### FgPfn contributes to microtubule organization

**Fig 7:**
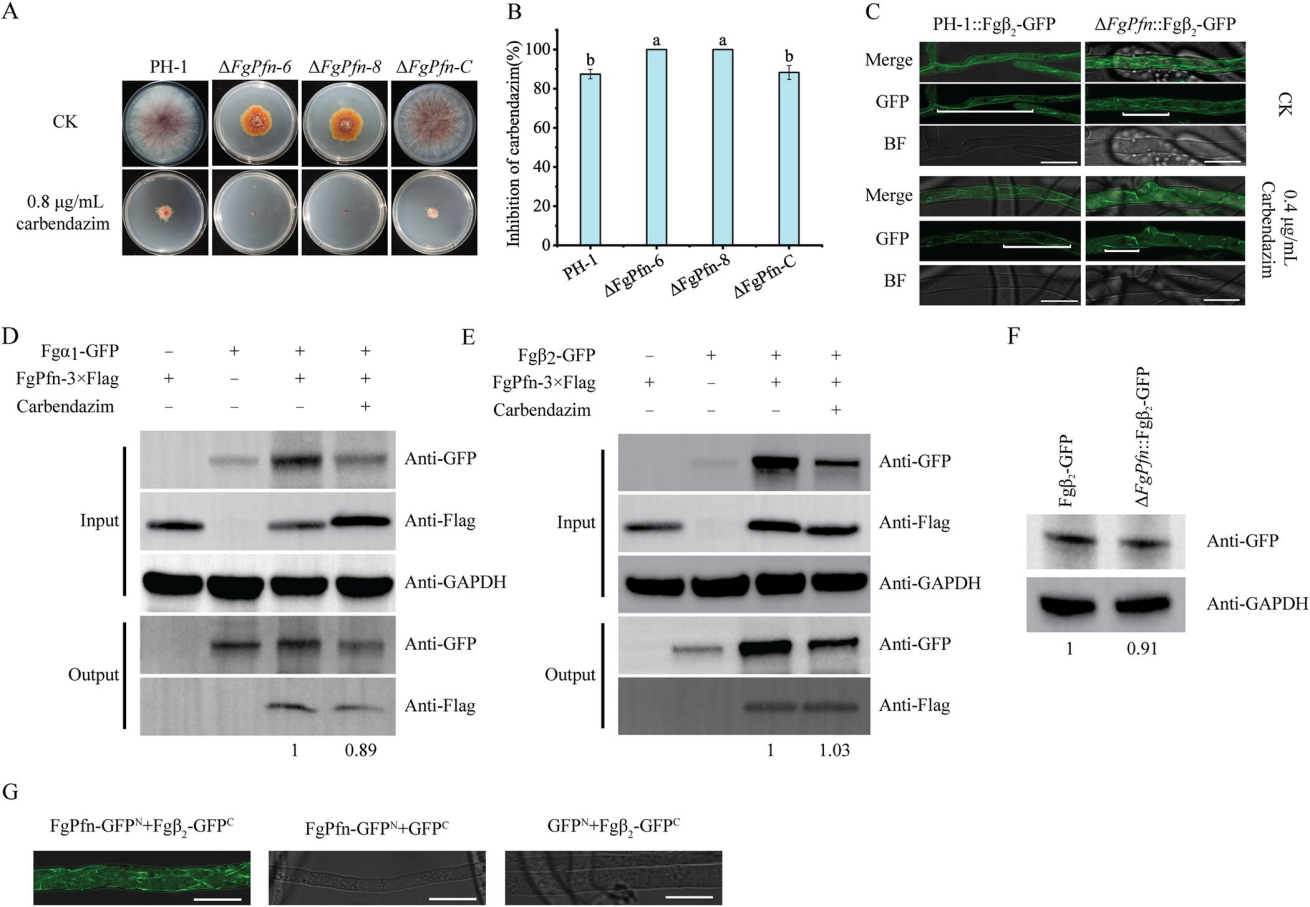
FgPfn regulates the sensitivity to carbendazim and contributes to the organization of microtubules. (A) Δ*FgPfn* mutants significantly increased the sensitivity to carbendazim. PH-1 and Δ*FgPfn*-C strains were able to grow after the 3d of 0.8 μg/mL carbendazim treatment, while the Δ*FgPfn* mutants were still unable to grow at 20d. (B) Mycelial growth inhibition of each strain by carbendazim quantified. (C) FgPfn contributes to microtubule organization. 10^5^ /mL conidia were inoculated in YEPD medium for 12h at 25°C, and then carbendazim with a final concentration of 0.4 μg/mL was added for another 12h. white lines indicate differences. Microtubule organization was examined under a confocal microscope. Bar = 10 μm. (D) Analysis of the interaction of FgPfn with Fgα_1_ by Co-IP assay. The Fgα1-GFP fusion plasmid was transformed into PH-1 and Δ*FgPfn*-C containing a 3×Flag tag, respectively. To explore the effect of carbendazim on the interaction, strains were cultured in YEPD 36h, and carbendazim with a final concentration of 2 μg/mL was added for another 12h. Total protein samples were first incubated with anti-GFP agarose beads (GFP-Trap Magnetic Agarose beads, ChromoTek, Redmond, WA, USA) following the manufacturer’s protocol. Then, 10 μL protein samples eluted from beads were analyzed by western blot. (E) Analysis of the interaction of FgPfn with Fgβ_2_ by Co-IP assay. The Fgβ_2_-GFP fusion plasmid was transformed into PH-1 and Δ*FgPfn-C* containing a 3×Flag tag, respectively. To explore the effect of carbendazim on the interaction, strains were cultured in YEPD 36h, and carbendazim with a final concentration of 2 μg/mL was added for another 12h. Total protein samples were first incubated with anti-GFP agarose beads following the manufacturer’s protocol. Then, 10 μL protein samples eluted from beads were analyzed by western blot. (F) The content of the Fgβ_2_-GFP protein in both PH-1 and Δ*FgPfn* mutant each strain was determined by western blot assay with the anti-GFP antibody. The protein samples were also detected with anti-GAPDH antibody as a reference. (G) Analysis of the interaction of FgPfn with Fgβ_2_ by BiFC assay. The strains bearing a single construct (FgPfn-GFP^N^ with GFP^C^ or GFP^N^ with Fgβ_2_-GFP^C^) were used as the negative control. The GFP signals in the hyphae of each strain were examined under a confocal microscope. Bar = 10 μm.

Benzimidazole fungicides carbendazim, which comprises a well-known group of β-tubulin interferers, has been extensively used to control various plant diseases caused by fungi. Furthermore, the mutations in*β*_*2*_*-tubulin* gene can affect the sensitivity of *F*. *graminearum* to carbendazim [41]. Here, we found that Δ*FgPfn* had significantly increased susceptibility to carbendazim. Under the treatment of 0.8 μg/mL carbendazim, the inhibition rates of PH-1 and Δ*FgPfn-C* were 85.21% and 84.63%, respectively, but the inhibition rates of Δ*FgPfn* deletion mutants were 100% (Fig 7A and 7B). To explore whether the change in carbendazim susceptibility was related to microtubules, the formation of microtubules in the mutant was further investigated with the strains expressing Fgβ_2_-GFP, a significant component of the microtubule. Compared with PH-1, Fgβ_2_-GFP in the Δ*FgPfn* could still be assembled into microtubules but were shorter (Fig 7C), and the content of Fgβ_2_-GFP in Δ*FgPfn* was decreased (Fig 7F). Furthermore, under the treatment of 0.4 μg/mL carbendazim, the organization of microtubules in the Δ*FgPfn* was strongly inhibited (Fig 7C). These findings suggested that FgPfn was required for normal organization of microtubules.

To further explore the regulatory mechanism of FgPfn on microtubule organization, we verified the interaction between FgPfn and Fgβ_2_ by Co-immunoprecipitation (Co-IP) assay and Bimolecular fluorescence complementation (BiFC) assay (Fig 7E and 7G). We also verified the interaction between FgPfn and Fgα_1_, a significant component of the microtubule, by Co-IP assays (Fig 7D). In addition, the effect of carbendazim on the interactions of FgPfn with Fgα_1_ and Fgβ_2_ was weak. To put it in a nutshell, we could conclude that FgPfn contributes to microtubule organization by interacting with Fgα_1_ and Fgβ_2_, thereby affecting the sensitivity of *F*. *graminearum* to carbendazim.

### FgPfn is required for FgMyo5-FgAct cytoskeleton organization

**Fig 8:**
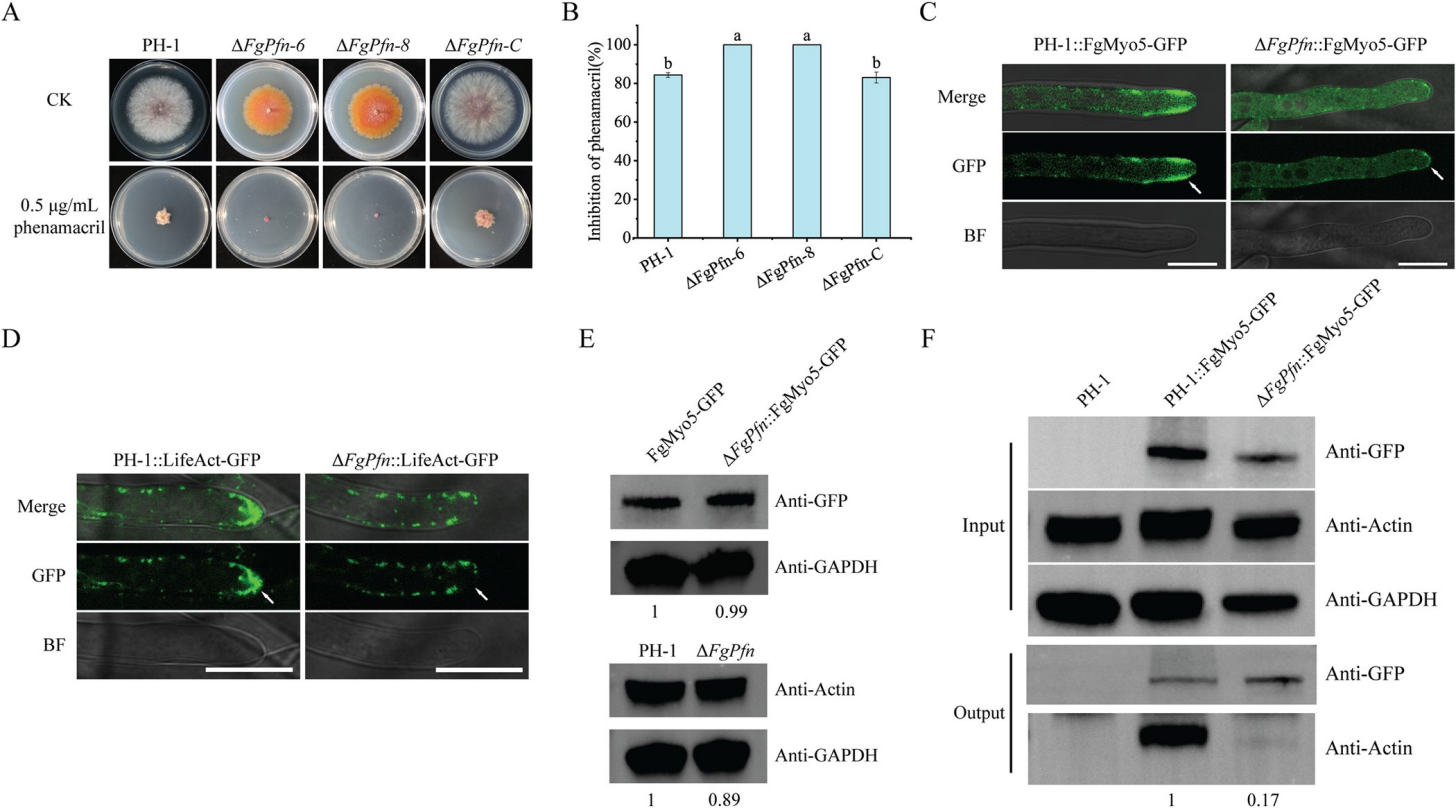
FgPfn is required for the FgMyo5-FgAct cytoskeleton organization. (A) Δ*FgPfn* mutants significantly increased the sensitivity to phenamacril. PH-1 and Δ*FgPfn*-C grew for the 3d under 0.5 μg/mL phenamacril treatment and the Δ*FgPfn* mutants grew for the 20d. (B) Mycelial growth inhibition of each strain by phenamacril quantified. (C) Deletion of *FgPfn* affected the FgMyo5 localization. The localization of FgMyo5 was observed by expressing FgMyo5-GFP in the wild-type and mutant strains. All strains were grown in YEPD for 12h and photographed under a confocal microscope. White arrows indicate differences. Bar = 10 μm. (D) Deletion of *FgPfn* affected the actin organization. Actin cable and patch were observed by expressing LifeAct-GFP in the wild-type and mutant strains. All strains were grown in YEPD for 12h and photographed under a confocal microscope. White arrows indicate differences. Bar = 10 μm. (E) The content of the FgMyo5 and FgAct in both PH-1 and Δ*FgPfn* mutant was determined by western blot assay with the anti-GFP antibody and anti-Actin antibody. The protein samples were also detected with anti-GAPDH antibody as a reference. (F) Analysis of the interaction of FgMyo5 with FgAct in Δ*FgPfn*. The FgMyo5-GFP fusion plasmid was transformed into PH-1 and Δ*FgPfn*, respectively. Total protein samples were first incubated with anti-GFP agarose beads following the manufacturer’s protocol. Then 10 μL protein samples eluted from beads were analyzed by western blot.

In this study, we also found Δ*FgPfn* increased the sensitivity to phenamacril, an effective fungicide in controlling FHB in past years. The Δ*FgPfn* mutants showed 100% inhibition rates under 0.5 μg/mL phenamacril treatment for 20d, but PH-1 could still grow with an 87.40% inhibition rate (Fig 8A and 8B). Similarly, Δ*FgPfn* from the highly phenamacril-resistant strain YP-1 also showed increased susceptibility to phenamacril (S3 Fig). Previous studies have shown that phenamacril acts on type I myosin FgMyo5 of *F*. *graminearum* and inhibits hyphal growth by inhibiting the ATPase activity of FgMyo5 and blocking the binding of FgMyo5 to FgAct [35–37]. Therefore, we wanted to know whether FgPfn affected FgMyo5. First, we constructed PH-1::FgMyo5-GFP mutant and Δ*FgPfn*::FgMyo5-GFP mutant for the FgMyo5 localization assay. The subcellular localizations of FgMyo5-GFP in the tip were disrupted in the Δ*FgPfn* mutant (white arrows) compared with PH-1 (Fig 8C). The actin at the tips of pathogenic filamentous fungal hyphae was involved in the completion of vigorous exocytosis activities [4] and responsible for polarized growth and infection [11,42]. We further verified whether FgPfn loss affects actin organization at the tip. Surprisingly, the subcellular localization of actin patches in the tip was utterly disrupted in the Δ*FgPfn* deletion mutant (Fig 8D), which was consistent with that in FgMyo5-GFP. Although the deletion of *FgPfn* caused severe defects in the FgMyo5-FgAct cytoskeleton, the effect on FgMyo5 and FgAct expression was weak (Fig 8E). However, the deletion of *FgPfn* reduced the strength of the interaction between FgMyo5 and FgAct (Fig 8F). This suggested that the deletion of *FgPfn* disrupted the FgMyo5-FgAct cytoskeleton organization and affected sensitivity to phenamacril.

**Fig 9:**
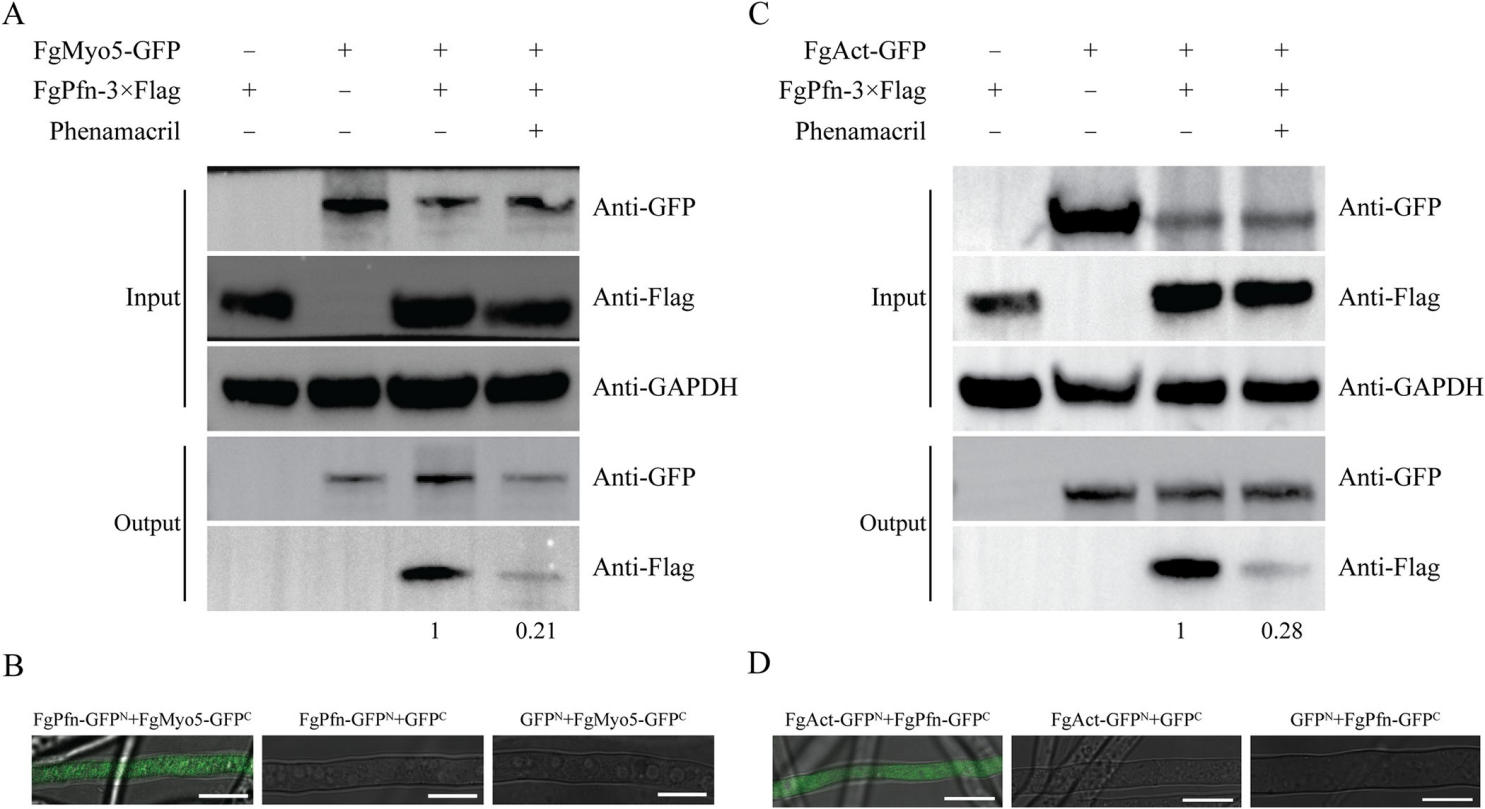
FgPfn interacts with both FgMyo5 and FgAct. (A) Analysis of the interaction of FgPfn with FgMyo5 by Co-IP assay. The FgMyo5-GFP fusion plasmid was transformed into PH-1 and Δ*FgPfn-C* containing a 3×Flag tag, respectively. To explore the effect of phenamacril on the interaction, strains were cultured in YEPD 36h, and phenamacril with a final concentration of 2 μg/mL was added for another 12h. Total protein samples were first incubated with anti-GFP agarose beads following the manufacturer’s protocol. Then, 10 μL protein samples eluted from beads were analyzed by western blot. (B) Analysis of the interaction of FgPfn with FgMyo5 by BiFC assay. The strains bearing a single construct (FgPfnt-GFP^N^ with GFP^C^ or GFP^N^ with FgMyo5-GFP^C^) were used as the negative control. The GFP signals in the hyphae of each strain were examined under a confocal microscope. Bar = 10 μm. (C) Analysis of the interaction of FgPfn with FgAct by Co-IP assay. The FgAct-GFP fusion plasmid was transformed into PH-1 and Δ*FgPfn-C* containing a 3×Flag tag, respectively. To explore the effect of phenamacril on the interaction, strains were cultured in YEPD 36h, and phenamacril with a final concentration of 2 μg/mL was added for another 12h. Total protein samples were first incubated with anti-GFP agarose beads following the manufacturer’s protocol. Then, 10 μL protein samples eluted from beads were analyzed by western blot. (D) Analysis of the interaction of FgPfn with FgAct by BiFC assay. The strains bearing a single construct (FgAct-GFP^N^ with GFP^C^ or GFP^N^ with FgPfn-GFP^C^) were used as the negative control. The GFP signals in the hyphae of each strain were examined under a confocal microscope. Bar = 10 μm.

To further confirm the regulatory mechanism of FgPfn on FgMyo5, Co-IP and BiFC assays were performed to verify the interaction between FgPfn and FgMyo5. The results showed that there was an interaction between FgPfn and FgMyo5 (Fig 9A and 9B). Here, we also found that there was a direct physical interaction between FgPfn and FgAct by Co-IP, BiFC and yeast two-hybrid (Y2H) assays (Figs 9C, 9D and S2B). In addition, the interaction strength of FgPfn with FgMyo5 and FgAct was reduced after phenamacril treatment. In conclusion, we found that FgPfn interacted with FgMyo5 and FgAct to regulate the FgMyo5-FgAct cytoskeleton organization and sensitivity to phenamacril.

### The protein integrity is necessary for FgPfn interaction with FgAct

**Fig 10:**
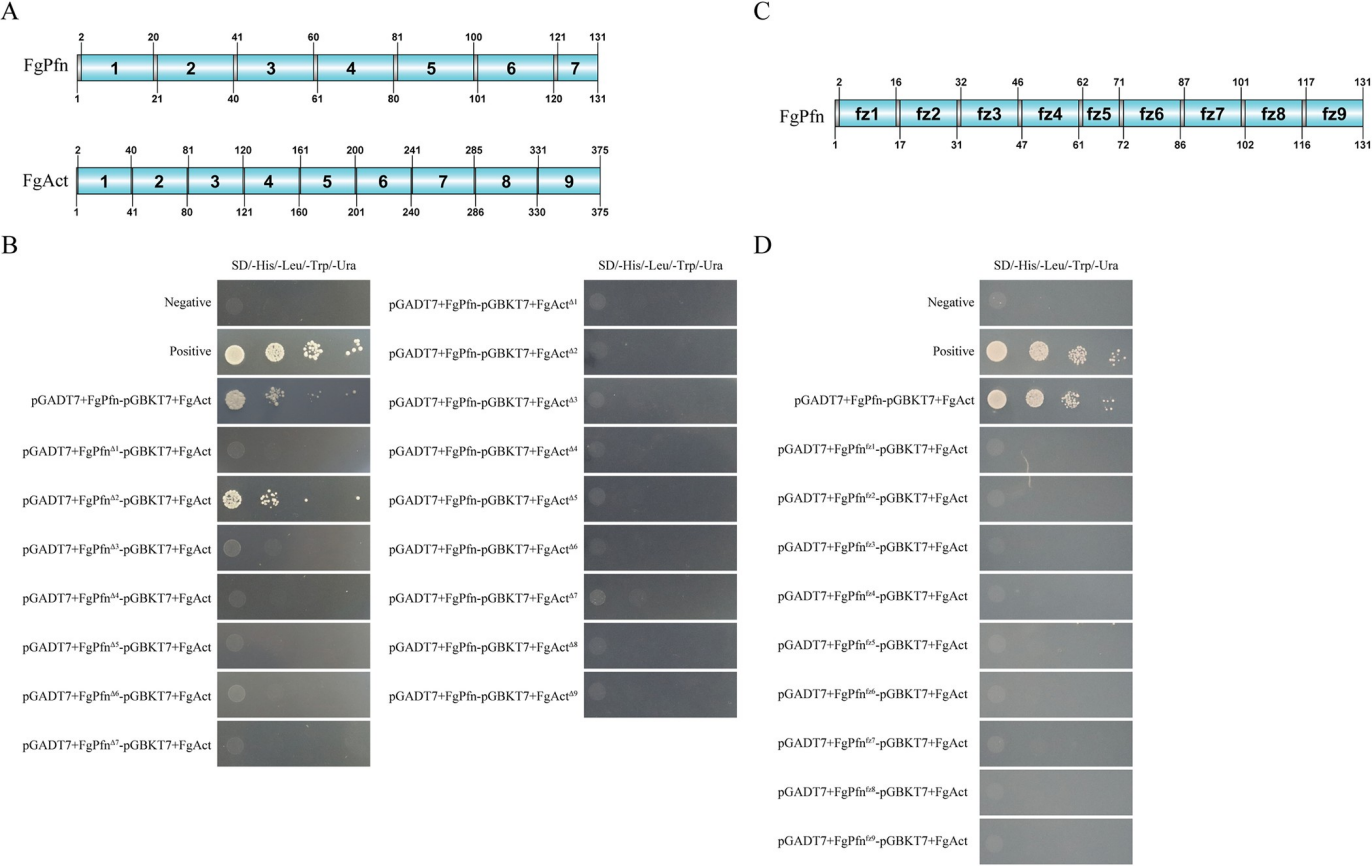
The protein integrity is necessary for FgPfn interaction with FgAct. (A) The subcloning motifs of FgPfn and FgAct were indicated. FgPfn and FgAct were divided into 7 and 9 motifs, respectively. Each cDNA of *FgPfn*, which lacked one motif, was cloned into pGADT7, and each cDNA of *FgAct*, which lacked one motif, was cloned into pGBKT7 plasmid, respectively. (B) Analysis of the interaction of FgPfn and FgAct subclone motifs. (C) The amino acid reversal mutations on FgPfn were indicated. FgPfn were divided into 9 motifs, reversed the order of the amino acid sequence of each segment respectively, and then cloned the 9 mutations of FgPfn into pGADT7 plasmid to confirm interaction with FgAct by Y2H. (D) Analysis of the interaction of FgPfn amino acid reversal mutations with FgAct by Y2H assay.

In eukaryotes, profilins regulate the cytoskeleton through the interaction between actin and related proteins, which is essential for maintaining normal life activities in the organism. We want to know whether one or more motifs are central to FgPfn-FgAct interaction systems. FgPfn and FgAct were divided into several motifs and subcloned into pGADT7 (yeast GAL4 activation domain vector) and pGBKT7 (yeast GAL4-binding domain vector) vectors, respectively. The effect of FgPfn and FgAct motifs deletion on FgPfn-FgAct interaction was confirmed by Y2H (Fig 10A and 10B). We found that only when FgPfn was divided into 7 motifs did the absence of motif 2 not affect the interaction with FgAct. All other conditions resulted in the non-interaction between FgPfn and FgAct. The results suggested that protein integrity was necessary for FgPfn-FgAct interaction.

In addition, we performed reverse mutation of the amino acid sequence on FgPfn with one motif mutation per 10 or 15 amino acids and verified the interaction between FgPfn mutations and the original FgAct by Y2H. The results showed that FgPfn with amino acid reversal mutations did not interact with FgAct any more (Fig 10C and 10D). These results suggested that protein integrity was necessary for the FgPfn-FgAct interaction.

### The critical residues of FgPfn involved in FgPfn-FgAct interaction

**Fig 11:**
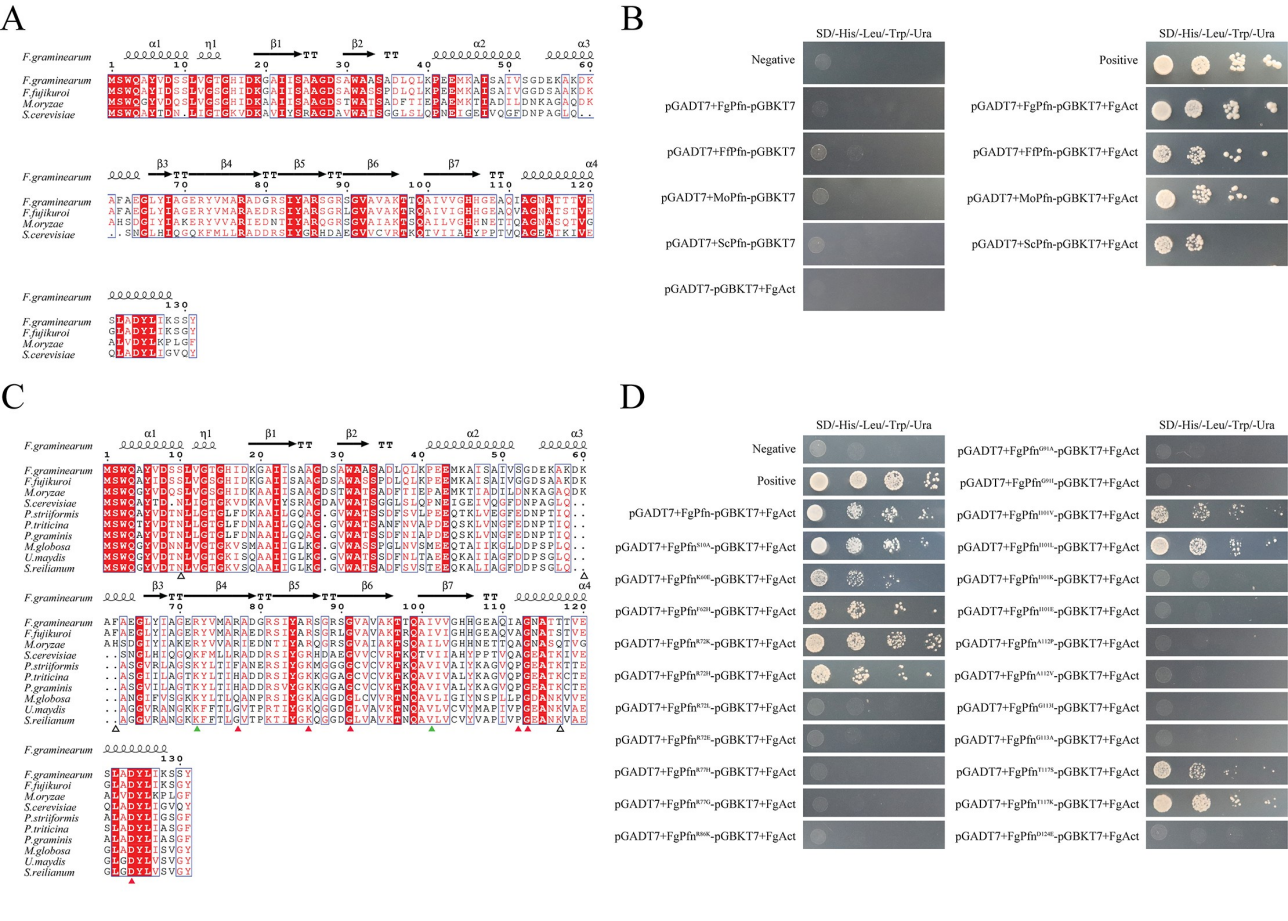
Sequence alignment and identification of conserved residues of profilins. (A) The sequence alignment of profilins among FgPfn, FfPfn, MoPfn, and ScPfn. The amino acids with the same polarity or acidity and basicity were annotated in red font. Identical amino acids were annotated with red shading. (B) Analysis of the interaction of FgPfn, FfPfn, MoPfn, and ScPfn with FgAct by Y2H assay. (C) The sequence alignment of profilins among ascomycetes and basidiomycetes. The amino acids with the same chemical properties were annotated in red font. Identical amino acids were annotated with red shading. (D) Analysis of the interaction of FgPfn amino acid mutations with FgAct by Y2H assay.

Although profilins as actin-binding proteins are conserved, the amino acid sequences vary among different species. Here, profilins in *Fusarium fujikuroi* (FfPfn), *Magnaporthe oryzae* (MoPfn), and *Saccharomyces cerevisiae* (ScPfn) were performed the amino acid sequences alignment compared with FgPfn. We found that among the 131 amino acid residues of FgPfn, there were 23 different amino acid residues, 67 residues with the same polarity or acidity and basicity (red font), and 41 conserved residues (red shading), accounting for 17.6%, 51.1% and 31.3%, respectively (Fig 11A). Then, the cDNA of *FfPfn*, *MoPfn*, and *ScPfn* were cloned into the pGADT7 vector and determined the interaction with FgAct by Y2H. Surprisingly, FfPfn, MoPfn, and ScPfn were all able to interact with FgAct (Fig 11B).

To further narrow down the conserved amino acid residues in profilins, we performed profilins sequence alignment from ascomycetes and basidiomycetes (Fig 11C). We found that among the 131 amino acid residues of FgPfn, there were 46 different amino acid residues, 59 residues with the same chemical properties (red font), and 26 conserved residues (red shading) for 35.1%, 45.0% and 19.8%, respectively. The conserved amino acids reduced from 41 (31.3%) to 26 (19.8%).

Based on the co-crystallization of profilins binding to actin in bovine, the amino acids in contact with actin in profilins and their functions have been analyzed [43,44]. On this basis, we further screened potential conserved amino acids involved in profilins-actin binding from conserved residues in *F*. *graminearum*. The residues K60, F62, R72, R76, R86, G91, A112, G113, T117 located on the profilins-actin binding surface and S10, I101, D124 far away from the binding surface, were selected to verify the effects on the interaction between FgPfn and FgAct by Y2H.

We mutated the screened amino acid residues into other residues with the same or different chemical properties. Then, we verified the impact of amino acid mutations on the interaction between FgPfn and FgAct by Y2H (Fig 11D). It was found that the S10A, K60E, F62H, and T117K (mutations of different chemical properties) mutations did not affect the FgPfn-FgAct interaction, indicating that S10, K60, F62, and T117 contributed less to the interaction between FgPfn and FgAct. We found that mutations of R72K/H and I101V/L (with the same chemical properties) did not affect the FgPfn-FgAct interaction. In contrast, mutations of R72L/E and I101K/E (mutations of different chemical properties) resulted in no interaction. The results above indicated that R72 and I101 were necessary for the FgPfn-FgAct interaction but could be replaced by other amino acids with the same polarity. In addition, mutations of R77, R86, G91, A112, G113, and D124 all resulted in the non-interaction between FgPfn and FgAct, indicating they are irreplaceable for the FgPfn-FgAct interaction.

### Conserved amino acids were required for the function of FgPfn

**Fig 12:**
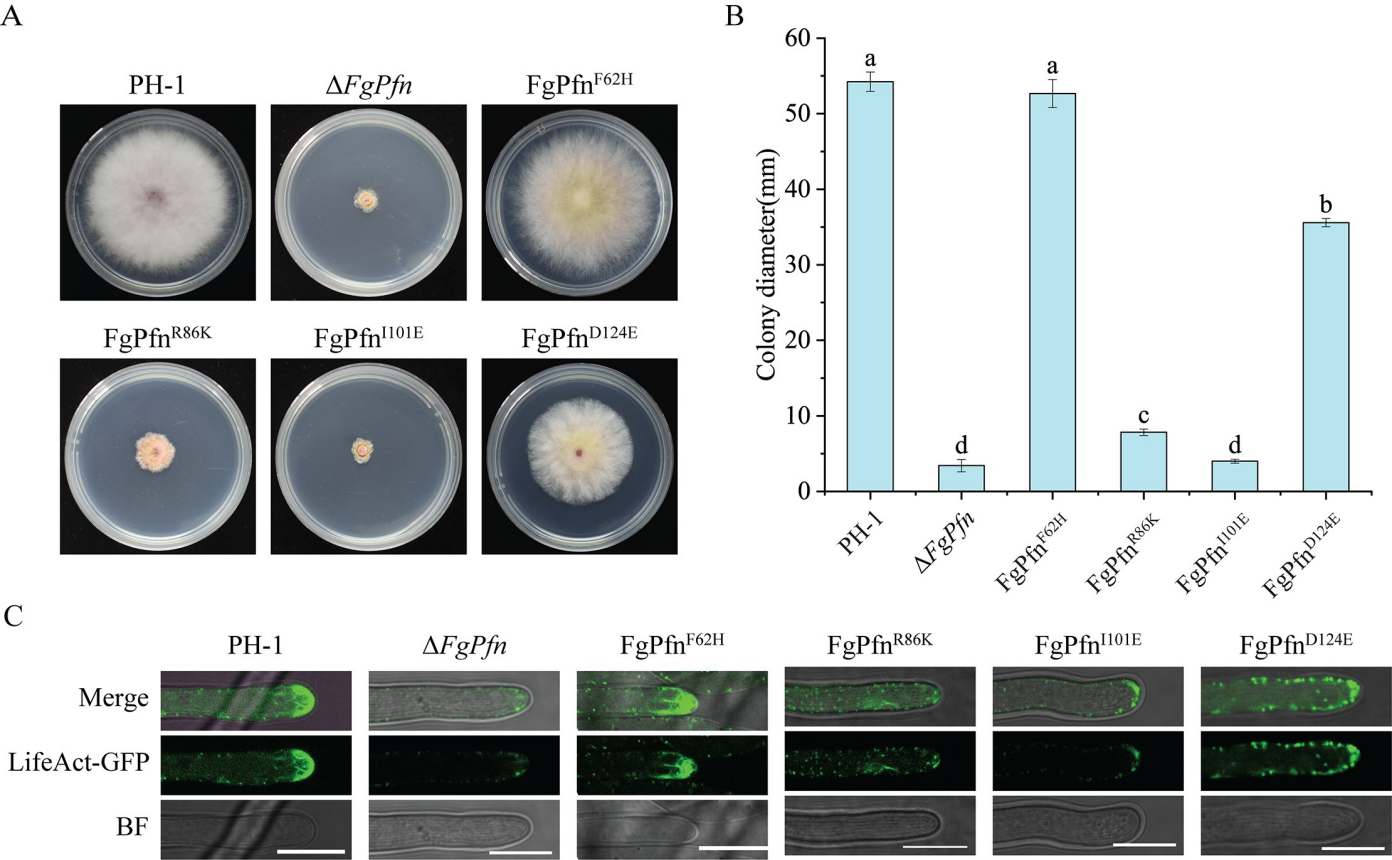
Conserved amino acids were required for the function of FgPfn. (A) Growth phenotypes of different mutants. Mycelial growth was photographed after the 3d culture of each mutant on PDA. (B) The colony diameter of each mutant strain was measured after the 3d growth on PDA. Bars with the same letter indicate no significant difference according to the LSD test at p < 0.05. (C) Mutations in conserved residues caused defects in actin organization. The images of actin organization were taken after the 24h culture of each mutant in YEPD. Bar = 10 μm.

Although we have identified conserved residues involved in the interaction between FgPfn and FgAct by Y2H, it is uncertain whether these residues contribute to actin organization. Three conserved residue mutations of R86K, I101E, D124E and a non-conserved residue mutation of F62H were selected to further explore the effects on actin organization. Amino acid mutation strains were constructed by PH-1::LifeAct-GFP, and were obtained at least two that were sequenced. The results showed that except for F62H mutation, all other mutations caused varying degrees of development defects (Figs 12A, 12B and S5). Also, the subcellular localizations of actin patches in the tip were impaired in conserved amino acid mutation strains (Fig 12C). Among them, the actin organization in I101E mutant was seriously disrupted, similar to that observed in the *FgPfn* deletion mutant.

Mutations in conserved residues also caused defects in asexual development. Compared with PH-1, the I101E mutation strain had severe defects in producing conidia with significantly reduced conidiation (S2 Table). In contrast, strains of the remaining three mutations showed no remarkable change in conidiation. However, except for F62H, these mutants exhibited a decrease in conidia length and a significant decrease in septa (S5E, S5F and S5G Fig), but the germination rate of different mutants was not affected (S2 Table). Mutations in conserved residues of FgPfn also resulted in reduced pathogenicity and DON content (S6 Fig). The above results indicated that the conserved interacting residues were crucial for the function of FgPfn. The mutations in conserved residues disrupt the interaction between FgPfn and FgAct, causing defects in actin organization, development and pathogenicity.

## Discussion

The cytoskeleton is a highly dynamic structure, so the rapid and correct assembly of actin is critical for completing various cellular life activities [1,2]. Profilin, an actin-binding protein, is essential in all organisms through its ability to interact with several proteins and link signaling pathways to the actin cytoskeleton, regulating actin depolymerization balance [22,23,26,45]. In this study, we identified the direct physical interaction of FgPfn with FgMyo5 and FgAct (Figs 9 and S2B), the targets of the fungicide phenamacril [35–37]. A series of truncations at the C terminus of profilin in yeast caused its functional defects [46]. The motifs mutation in FgPfn suggested that protein integrity was necessary for FgPfn-FgAct interaction (Fig 10). Sequence alignment of profilins from Ascomycota and Basidiomycota was further performed and amino acid residues in FgPfn that may contribute to actin organization were identified (Fig 11). These amino acids may be binding sites for FgPfn-FgAct interactions, stabilize FgPfn-FgAct interactions by forming salt bridges, or play an essential role in maintaining the dimensional structure of FgPfn protein [44].

Previously, mutations in profilin were found to cause deficiencies in organisms by affecting actin. For example, the C71G, M114T, G118V and H120E mutations display reduced levels of bound actin [47]. The T109M mutation abrogates a phosphorylation site in profilin 1 [48]. The A20T and Q139L [49], C71G [50] mutations form insoluble aggregates. These mutations could disrupt the actin filament organization mechanism, leading to human Amyotrophic Lateral Sclerosis (ALS) disease [51–53]. Profilin mutations in yeast resulted in lower actin affinity and defects in growth, fluid-phase endocytosis, and actin organization [44,53–55]. Interestingly, the previously reported mutations of profilin affecting profilin-actin interaction all fall within our putative conserved amino acids [44,54,55] (Fig 11). However, studies of profilin functions in *vivo*, especially in filamentous fungi, are lacking. The deletion of *FgPfn* significantly disrupted the organization FgMyo5 and actin in the tip of mycelium (Fig 8C and 8D) and reduced the interaction between FgMyo5 and FgAct (Fig 8F). Further experiments confirmed that conserved residues in FgPfn were crucial for the FgPfn-FgAct interaction. The R86K, I101E and D124E mutations caused varying degrees of defects in actin organization (Figs 11 and 12). Our results indicate that FgPfn is critical for actin organization and mutations in conserved amino acids result in actin organization failure. Current studies show that phenamacril acts on I-type myosin FgMyo5, inhibiting the ATPase activity of FgMyo5 and blocking the binding of FgMyo5 to FgAct [35–37]. The disrupted localization patterns of FgMyo5 affected the sensitivity to phenamacril [56]. The interaction of FgPfn with FgMyo5 and FgAct was reduced by phenamacril treatment (Fig 9A and 9C) and the deletion of *FgPfn* significantly increased the sensitivity to phenamacril (Figs 8A, 8B and S3). This indicated that FgPfn regulated the actin cytoskeleton by participating in FgMyo5-FgAct organization and interaction.

Increasing evidence suggests that in addition to binding actin, profilins also bind to specific polyproline-rich regions in other actin-regulated proteins, and serve as hubs for controlling complex molecular interaction networks [18], such as formins and Srv2 [19,20]. Recently, new research has found that profilins promote the coordination of actin and microtubule systems and modulate microtubule dynamics formins-mediated indirect interaction [30]. However, there is a lack of in *vivo* studies on the effects of profilins on microtubules. In this study, we found a physical interaction of FgPfn with Fgα_1_ and Fgβ_2_ (Fig 7D and 7E). The bimolecular fluorescence complementation (BiFC) assay indicated that FgPfn and Fgβ_2_ were close to each other (Fig 7G), while FgPfn was far from Fgα_1_ (S2A Fig). We speculated that after forming a complex with formins [30], FgPfn binds to Fgα1, while it can directly combine with Fgβ_2_ alone. Profilins increase the rate of microtubule growth several-fold in *vitro* systems, but microtubule formation persists in the absence of profilins [21]. Here, the content of Fgβ_2_-GFP in Δ*FgPfn* was comparable (Fig 7F), and microtubules could still be assembled (Fig 7C). However, Δ*FgPfn* only formed shorter microtubules, and the microtubule organization was strongly inhibited by carbendazim (Fig 7C). Actin filaments and microtubules are essential cytoskeletal proteins and exhibit extensive crosstalk in organization and function [57]. Complete actin organization defects caused by loss of FgPfn may contribute to microtubule organization defects. Furthermore, there is evidence that toxisomes are associated with the network of microtubules, and the integrity of the microtubules network is essential for toxisome assembly [56,58].

Deoxynivalenol (DON) is a critical virulence factor in *F*. *graminearum* [38]. DON biosynthetic organelle, the toxisome, is a remodeled perinuclear endoplasmic reticulum (ER) modified by the FgMyo5-FgAct cytoskeleton and microtubules in *F*. *graminearum* [38,58,59], inhibiting FgMyo5, FgAct and microtubules all lead to disrupts the toxisome formation [38,39,60]. The actin capping proteins (CAPs) FgCapA/B interact with actin, and the deletion of *FgCapA* or *FgCapB* results in defective toxisome formation, DON production and virulence [12]. Microtubule organization exhibited unstable organization in the absence of Fgα_1_/β_2_ and microtubule end-binding protein FgEB1, resulting in disrupted DON production and toxisome formation [56,58]. We found actin accumulated around the toxisomes in wild type induced by trichothecene biosynthesis induction (TBI) medium (Fig 6C). However, that failed to accumulate in the Δ*FgPfn*, which resulted in defective toxisome formation (Fig 6A and 6D), suggesting that FgPfn was necessary for the toxisome formation. The trichothecene efflux pump Tri12 interacts with toxisomes and participate in DON transport through actin cytoskeletal-dependent vesicles and vacuoles [34,40]. However, there was no fluorescence signal in the Δ*FgPfn*::Tri12-GFP strain (Fig 6B). Actin at the tips of pathogenic filamentous fungal hyphae was responsible for polarized growth and infection [11,42]. Here, we found that the subcellular localization of actin in the tip was completely disrupted in the Δ*FgPfn* mutant. At the same time, defects in microtubule organization in Δ*FgPfn* also contribute to reduced virulence. Thus, the reduced virulence of Δ*FgPfn* is due to impaired growth, reduced DON production by toxisome formation defect, and DON transport due to FgPfn-dependent cytoskeletal disruption.

**Fig 13:**
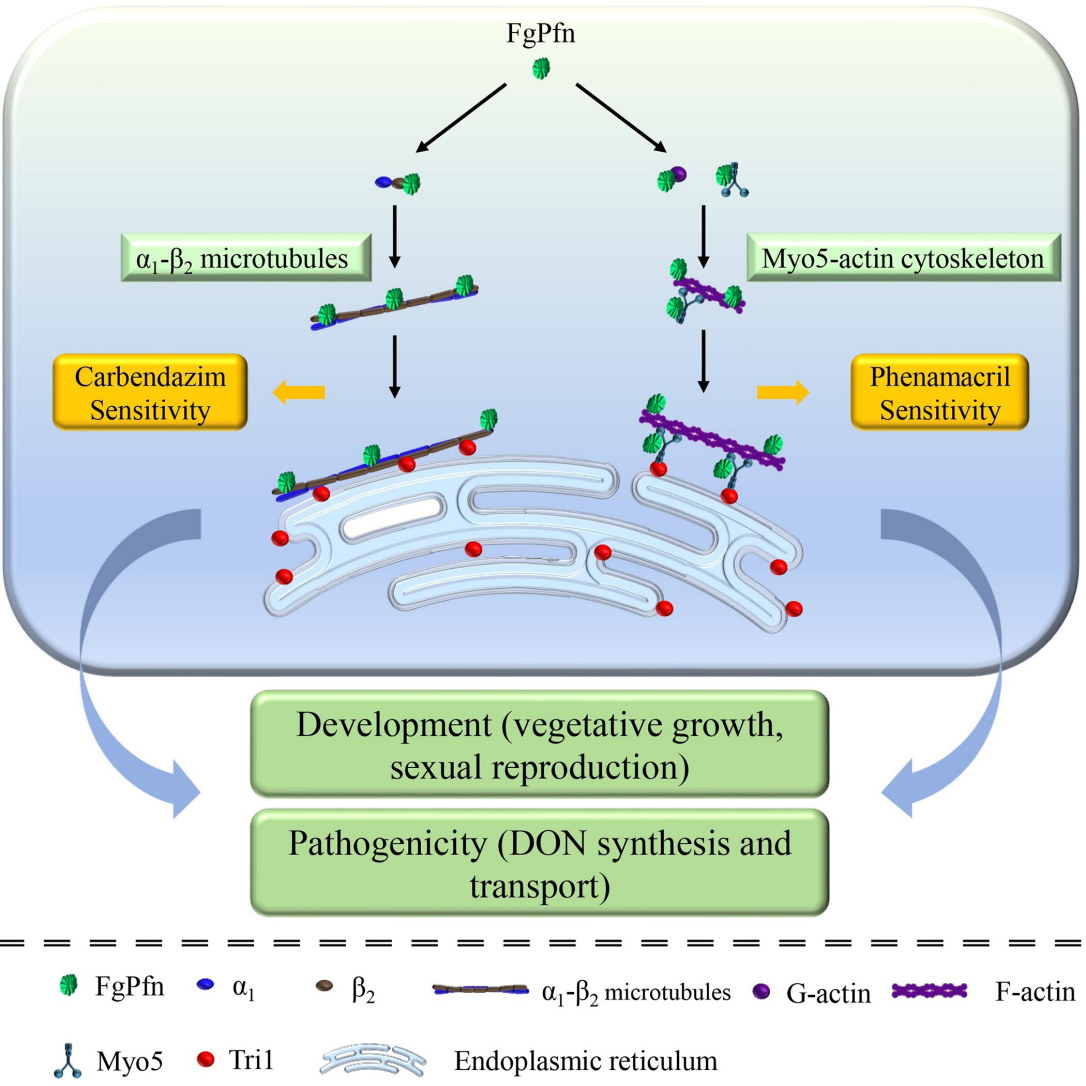
The schematic illustration of the interaction and function of FgPfn in FgPfn-dependent cytoskeleton. The interaction between FgPfn with Fgα_1_ and Fgβ_2_ contributes to microtubule organization and the sensitivity to carbendazim. FgPfn regulated the FgMyo5-FgAct cytoskeleton and the sensitivity to phenamacril by participating in the organization and interaction of FgMyo5 and FgAct. FgPfn regulates the formation of toxisomes by participating in the reorganization of actin induced in TBI. In this way, FgPfn regulates the vegetative growth, sexual reproduction and pathogenicity of the filamentous fungus *Fusarium graminearum*.

All evidence suggests that FgPfn is required with the microtubule and actin cytoskeleton. Deletion of *FgPfn* disrupts the FgPfn-dependent cytoskeleton, including defects in microtubule organization, FgMyo5-FgAct organization and interaction, resulting in subsequent developmental defects in *F*. *graminearum*, including reduced hyphal growth and conidiation, obstructed sexual reproduction and decreased pathogenicity, accompanied by marked disruptions in DON biosynthesis and transport. Therefore, we propose a model FgPfn is an essential cytoskeletal regulatory protein associated with both microtubules and actin, involved in the development and pathogenicity of *F*. *graminearum* (Fig 13).

## Material and methods

### Sequence analysis

Gene and amino acid sequences were retrieved from the fungal genome database FungiDB (https://fungidb.org/fungidb/). Structural domains were predicted online using SMART (https://smart.embl.de/) and the NCBI database (http://www.ncbi.nlm.nih.gov/Structure/cdd/wrpsb.cgi/). Amino acid sequences of other species were obtained for phylogenetic tree analysis using BLASTP (https://BLAST.ncbi.nlm.nih.gov/BLAST.cgi). Sequence alignment was performed with the ClustalW program. Maximum Likelihood (ML) method, J Le and Gascuel (LG) model and 1000 replicates were used to construct the phylogenetic tree by MEGA7. The domains were drawn with the Batch SMART in TBtools (https://github.com/CJ-Chen/TBtools/releases).

### Strains, culture, and fungicide assay

The strains used in this study are listed in S1 Table. The wild-type *F*. *graminearum* strain PH-1 was used as the parental strain to construct the derived mutants in this study. All strains were cultured on PDA with 3-mm mycelial plugs at 25°C. Mycelial growth of wild-type, deletion mutants and mutations were assayed on potato dextrose agar (PDA), complete medium (CM), minimum medium (MM), and V8 medium. To determine the sensitivity of deletion mutant to phenamacril (95%, Jiangsu Pesticide Research Institute Co., Ltd.) and carbendazim (98%, Jiangsu Rotam Chemistry Co., Ltd), we measured the inhibition rate of mycelial growth at the same concentration (phenamacril 0.5 μg/mL, carbendazim 0.8 μg/mL). To assay stress sensitivities, 3-mm mycelial plugs were inoculated on PDA supplemented with 1.2 M NaCl, 1.2 M KCl, 0.05% SDS, 0.03% Congo Red, and 1.2 M Sorbitol, and PDA plate without added any stresses factor was used as a control. Inhibition rates = (Average diameters of control group—Average diameters of treatment group)/Average diameters of control group × 100%. All experiments were repeated three times independently.

### Mutant construction

Protoplast preparation and transformation were performed following previously reported protocols [61]. The open reading frame (ORF) of *FgPfn* (FGSG_06392) was replaced by the *HPH-HSV-tk* fragment to construct deletion mutants from wild type strain PH-1. Furthermore, all transformants were analyzed by polymerase chain reaction (PCR) and Southern blot. For complementation of the Δ*FgPfn* strain, the *FgPfn* gene with 3×Flag tag fusion vector was sequenced and transformed into the Δ*FgPfn* deletion mutant to construct the complementation mutant of Δ*FgPfn*. The primers used in this study are shown in S3 Table.

To construct the amino acid mutation vector, primers containing base mutations were used to amplify the fragment via PCR with Phanta Max Super-Fidelity DNA Polymerase (Vazyme, China) and were assembled with MultiF Seamless Assembly Mix (ABclonal, China) according to the manufacturer’s instructions. The products were appropriately diluted and then amplified via PCR. DNA fragments were confirmed by Sanger sequencing (Sangon, China)

For construction of FgPfn-GFP fusion cassette, the ORF of *FgPfn* without stop codon was amplified via PCR with Phanta Max Super-Fidelity DNA Polymerase. The PCR products and Xho1-digested pYF11 were assembled with MultiF Seamless Assembly Mix and directly transformed into *E*. *coli* DH5α chemically competent cells (Sangon, China) for plasmid maintenance. Plasmids and colonies were confirmed by Sanger sequencing. With a similar strategy, other GFP fusion vectors were also constructed. Using the same strategy, we constructed the LifeAct-GFP fusion cassette with LifeAct amino acid residues (MGVADLIKKFESISKEE). Then the vectors transformed into the corresponding strain.

The construction of mutation strains was achieved by utilizing primers containing mutant bases for PCR amplification of the corresponding fragment using Phanta Max Super-Fidelity DNA Polymerase. Subsequently, the point mutation fragment was ligated with the G418 fragment through MultiF Seamless Assembly Mix to generate recombinant fragments. The open reading frame (ORF) of the *FgPfn* in the wild-type strain PH-1 was then substituted with the recombinant fragments. The resulting transformants were subjected to validation through PCR and Southern blot analysis.

### Infection and DON production assays

Infection assays on flowering wheat heads and wheat coleoptiles were performed as previously described [62], injecting 10 μL 10^6^ /mL conidial suspension into flowering wheat heads or 2 μL 10^6^ /mL conidial suspension into wheat coleoptiles, and inoculated with the same volume of water as a control check. For deoxynivalenol (DON) production assays, all the strains were grown in DON-inducing trichothecene biosynthesis induction (TBI) medium at 28°C for 7d in the dark. DON was quantitatively measured using a competitive ELISA-based DON detection plate kit (Wise) and according to the previous study [39,62].

### Microscopic examinations

The mutants of PH-1::LifeAct-GFP, PH-1::FgMyo5-GFP, and Δ*FgPfn*::LifeAct-GFP, Δ*FgPfn*::FgMyo5-GFP were cultured in YEPD for 36h, and then the GFP fluorescence signals were observed with TCS SP8 confocal microscope (Leica). The mutants of PH-1::Fgβ_2_-GFP and Δ*FgPfn*::Fgβ_2_-GFP were observed as previously described [63]. The mutants of PH-1::Tri1-GFP and Δ*FgPfn*::Tri1-GFP were cultured in TBI for 36h, and then the GFP fluorescence signals were observed with TCS SP8 confocal microscope (Leica).

### Yeast two-hybrid (Y2H) assay

For Y2H assays, the coding sequences were amplified from the cDNA of PH-1 with the primers shown in S3 Table. pGADT7 (yeast GAL4 activation domain vector) and pGBKT7 (yeast GAL4-binding domain vector) (Clontech) were digested with the restriction enzyme EcoRI, and then the cDNA fragments were inserted into the corresponding vector with MultiF Seamless Assembly Mix and directly transformed into *E*. *coli* DH5α chemically competent cells for plasmid maintenance. A pair of plasmids were verified by sequencing and then transformed into *Saccharomyces cerevisiae* AH109 according to the lithium acetate/single-stranded DNA/polyethylene glycol transformation protocol [64]. In addition, pGBKT7-53 with pGADT7-T served as a positive control, and pGBKT7-Lam with pGADT7-T were used as a negative control. Transformants were grown on synthetic medium (SD) lacking Leu and Trp (SD/-Leu/-Trp) for 3d at 28°C and then transferred to SD lacking His, Leu, Trp, and Ura (SD/-His/-Leu/-Trp/-Ura). Three independent experiments were performed to confirm the Y2H assay results.

For subcloning of FgPfn and FgAct, FgPfn and FgAct were divided into 7 and 9 motifs respectively. Each cDNA of *FgPfn* which lacked one motif was cloned into pGADT7 and each cDNA of FgAct which lacked one motif was cloned into pGBKT7 plasmid respectively. The interaction of the FgPfn mutations with FgAct and the interaction of FgAct mutations with FgPfn were confirmed by Y2H.

For amino acid reversal mutations, FgPfn were divided into 9 motifs, reversed the order of the amino acid sequence of each segment respectively, and then cloned the 9 mutations of FgPfn into pGADT7 plasmid to confirm interaction with FgAct by Y2H.

For analysis of the interaction of FgPfn amino acid mutations with FgAct by Y2H assay, sequence alignment of profilins between Ascomycota and Basidiomycetes was first performed to obtain conserved amino acids. Then, combined with the reported FgPfn-FgAct binding model, conserved residues located at different binding positions were selected. The screened amino acid residues were mutated into other residues with the same or different chemical properties, and the effect of the amino acid mutations on the interaction between FgPfn and FgAct was verified by Y2H.

### Bimolecular fluorescence complementation (BiFC) assay

For BiFC assays, the tested gene containing the native promoter region and ORF was amplified via PCR with Phanta Max Super-Fidelity DNA Polymerase. pHZ65 (N-terminal part of GFP) and pHZ68 (C-terminal part of GFP) were digested with the restriction enzyme XhoI. Then the fragments were inserted into the corresponding vector with MultiF Seamless Assembly Mix and directly transformed into *E*. *coli* DH5α chemically competent cells for plasmid maintenance. A pair of plasmids were verified by sequencing and then transformed into PH-1 as described previously [58]. GFP signal in hyphae was examined with Leica TCS SP8 confocal microscope.

### Western blot and Co-immunoprecipitation (Co-IP) assay

All mutants were incubated in yeast exact peptone dextrose (YEPD) at 25°C for 36h, and after that, the mycelia were harvested for protein extraction. To explore the effect of fungicides on the interaction, strains were cultured in YEPD 36h, carbendazim or phenamacril with a final concentration of 2 μg/mL was added for another 12h. Protein extraction and western blot analysis were performed as previously described [39].

The FgMyo5-GFP, FgAct-GFP, Fgα_1_-GFP and Fgβ_2_-GFP fusion plasmid were transformed into PH-1 and Δ*FgPfn-C* containing a 3×Flag tag, respectively. Transformants were verified by western blot assay with the anti-Flag and anti-GFP antibodies respectively. Total protein samples were first incubated with anti-GFP agarose beads (GFP-Trap Magnetic Agarose beads, ChromoTek, Redmond, WA, USA) following the manufacturer’s protocol. Then 10 μL protein samples eluted from beads were analyzed by western blot. All experiments were repeated twice.

### Statistical analysis

All data were presented as mean ± standard deviation and analyzed using a one-way analysis of variance (DPS) followed by Fisher’s least significant difference (LSD) test, P < 0.05.

## Accession number

The GenBank accession numbers of the amino acid sequence data in this article are as follows: XP_011325054.1 *F*. *graminearum*, XP_009257229.1 *F*. *pseudograminearum*, XP_031045137.1 *F*. *oxysporum*, XP_018751983.1 *F*. *verticillioides*, XP_023427220.1 *F*. *fujikuroi*, XP_003711995.1 *P*. *oryzae*, XP_024545923.1 *B*. *cinerea*, XP_011394188.1 *N*. *crassa*, CBF86948.1 *A*. *nidulans*, XP_717614.1 *C*. *albicans*, NP_014765.3 *S*. *cerevisiae*, OAV95406.1 *P*. *triticina*, XP_003330410.1 *P*. *graminis*, XP_047801062.1 *P*. *striiformis*, XP_001732053.1 *M*. *globose*, XP_011389817.1 *U*. *maydis*, CBQ73706.1 *S*. *reilianum*, and NP_955378.1 *H*. *sapiens*.

## Supporting information

S1 Fig Construction of Δ*FgPfn* deletion mutant and amino acid mutation strains. (A) Construction method of *FgPfn* deletion mutant. (B) Construction method of *FgPfn* complementation mutant. (C) Confirmation of *FgPfn* deletion mutants and complementation mutant by Southern Blot. (D) Construction method of amino acid mutation strains. (E) Confirmation of amino acid mutation strains by Southern Blot.
(TIF)

S2 Fig Interaction analysis with FgPfn-related proteins. (A) Analysis of the interaction of FgPfn with Fgα_1_ by bimolecular fluorescence complementation (BiFC) assay. Bar = 10 μm.(B) Analysis of the interaction of FgPfn with FgAct, FgMyo5, Fgα_1_ and Fgβ_2_ by yeast two-hybrid (Y2H) assay. *Saccharomyces cerevisiae* AH109 strains containing the pGADT7 and pGBKT7 plasmid pairs can form colonies on SD-L-T medium, and the positive control and the interacting strains can form colonies on SD-H-L-T-U medium.
(TIF)

S3 Fig The sensitivity of Δ*FgPfn* to phenamacril. Δ*FgPfn-Y2*, from the highly phenamacril-resistant strain YP-1, also showed growing sensitivity to phenamacril compared to the parental strains and complementation strain.
(TIF)

S4 Fig Subcellular localization of FgPfn-GFP. The strain was grown in YEPD for 36h and photographed under a confocal microscope. Bar = 10 μm.
(TIF)

S5 Fig Conserved amino acids in FgPfn affected vegetative growth and the sensitivity to various stresses. (A) Growth phenotype of different mutants. The colony morphology was photographed after the 3d inoculation of each strain on PDA, CM, MM, and V8 medium. (B) The colony morphology of different mutants on different medium. PH-1 and the mutation strains were inoculated on a PDA medium containing 1.2 M NaCl, 1.2 M KCl, 0.05% SDS, 1.2 M Sorbitol, and 0.03% Congo red at 25°C. The colony morphology of PH-1, F62H, and D124E strains was photographed after the 3d of growth, while R72E and R86K were photographed after 10d, and I101E was photographed after 20d of growth. (C) The mycelial growth rate of mutants was measured after the 3d growth on PDA, CM, MM, and V8 mediums. Bars with the same letter indicate no significant difference according to the least significant difference (LSD) test at p < 0.05. (D) The inhibitory effects of different stress factors on the hyphal growth of different strains were assessed by calculating the inhibition rates. The colony diameters of the strains grown on a PDA medium were used as the control. Inhibition rates = (Average diameters of control group—Average diameters of treatment group)/Average diameters of control group × 100%. Bars with the same letter indicate no significant difference according to the LSD test at p < 0.05. (E) Mutations of FgPfn resulted in conidial morphological defects. Conidia were examined by DIC microscopy. The septum of conidia was stained with CFW and photographed with an inverted fluorescent microscope at 20×. The nuclei of conidia were stained with DAPI for 30min and photographed with an inverted fluorescent microscope at 20×. Bar = 10 μm. (F) The average conidia length of each strain was measured with 100 conidia, which was repeated three times. Bars with the same letter indicate no significant difference according to the LSD test at p < 0.05. (G) The number of conidial septa of each strain was counted after staining with CFW, and then the proportion of conidia with different septate numbers to the total number was calculated. Count 100 conidia for each strain and repeat three times. Bars with the same letter indicate no significant difference according to the LSD test at p < 0.05.
(TIF)

S6 Fig **Conserved residue mutations in FgPfn affected pathogenicity** (A) Pathogenicity assays of different mutants on wheat coleoptiles. Add 2 μL 1×10^6^ /mL conidia suspension was inoculated on the injured wheat coleoptile for 7d to observe the incidence and take photos. The wheat variety was Huaimai 33. (B) The average length of lesion on wheat coleoptile infected by each strain was measured after 7d post-inoculation. Bars with the same letter indicate no significant difference according to the LSD test at p < 0.05. (C) DON content assay of different mutants. After the 7d of TBI culture, the DON content in the wild-type PH-1 and mutation strains were determined. Bars with the same letter indicate no significant difference according to the LSD test at p < 0.05. (D) Relative gene expression level of *TRI1*, *TRI4*, *TRI5*, *TRI6* and *TRI12* in the strains tested. After the 36h culture in TBI, mycelia of each strain were harvested for RNA extraction. The *GAPDH* was used as a reference gene. Bars with the same letter indicate no significant difference according to the LSD test at p < 0.05.
(TIF)

S1 Table List of strains used in this study. (XLSX)

S2 Table Conidiation and conidia germination of amino acid mutations in FgPfn. (XLSX)

S3 Table List of primers used in this study. (XLSX)

S1 Data Excel includes values used to generate histograms and Integrated density. (XLSX)
