# Supplementary figures and images for: FgPfn participates in vegetative growth, sexual reproduction, pathogenicity, and fungicides sensitivity via affecting both microtubules and actin in the filamentous fungus Fusarium graminearum
Source: PLoS Pathog. 2024 May 3;20(5):e1012215. doi: 10.1371/journal.ppat.1012215 (PMC11095717; doi:10.1371/journal.ppat.1012215)

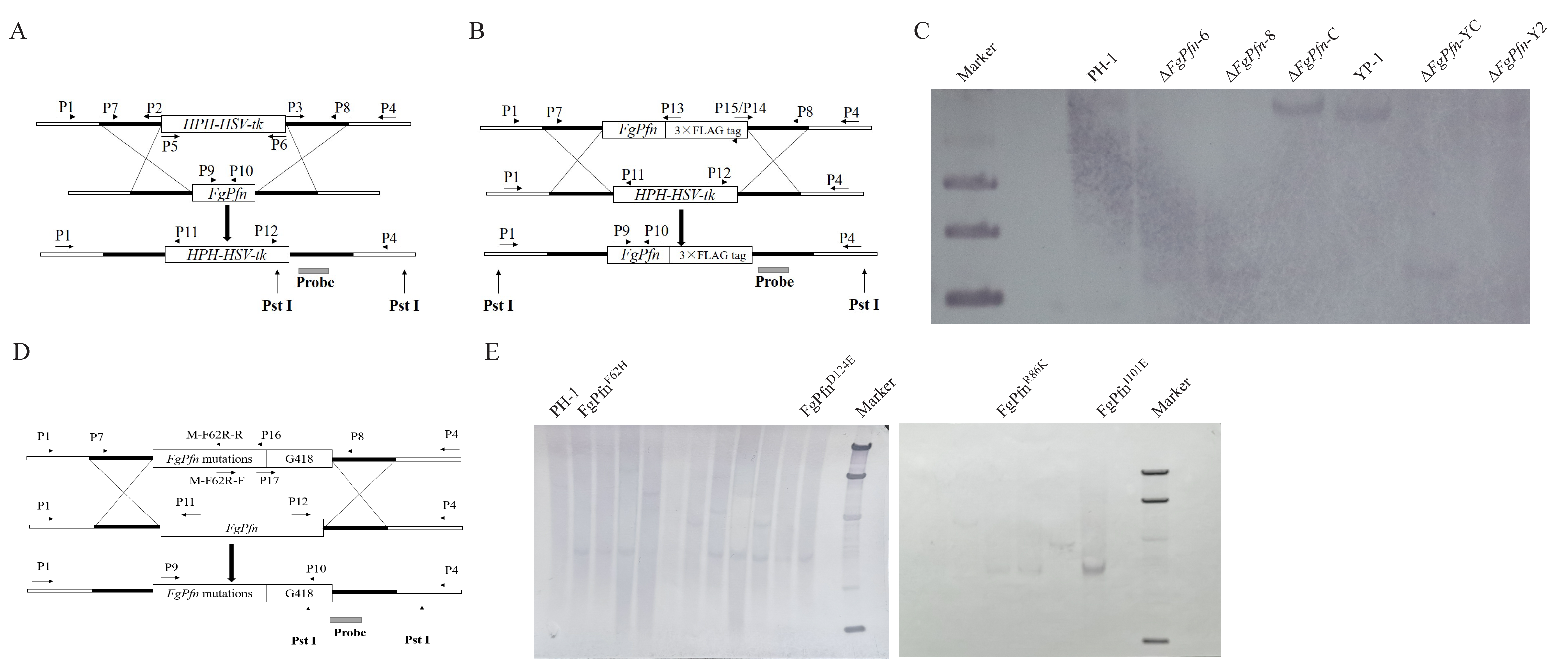

Supplement: S1 Fig — (A) Construction method of FgPfn deletion mutant. (B) Construction method of FgPfn complementation mutant. (C) Confirmation of FgPfn deletion mutants and complementation mutant by Southern Blot. (D) Construction method of amino acid mutation strains. (E) Confirmation of amino acid mutation strains by Southern Blot. (TIF) [file ppat.1012215.s001.tif]

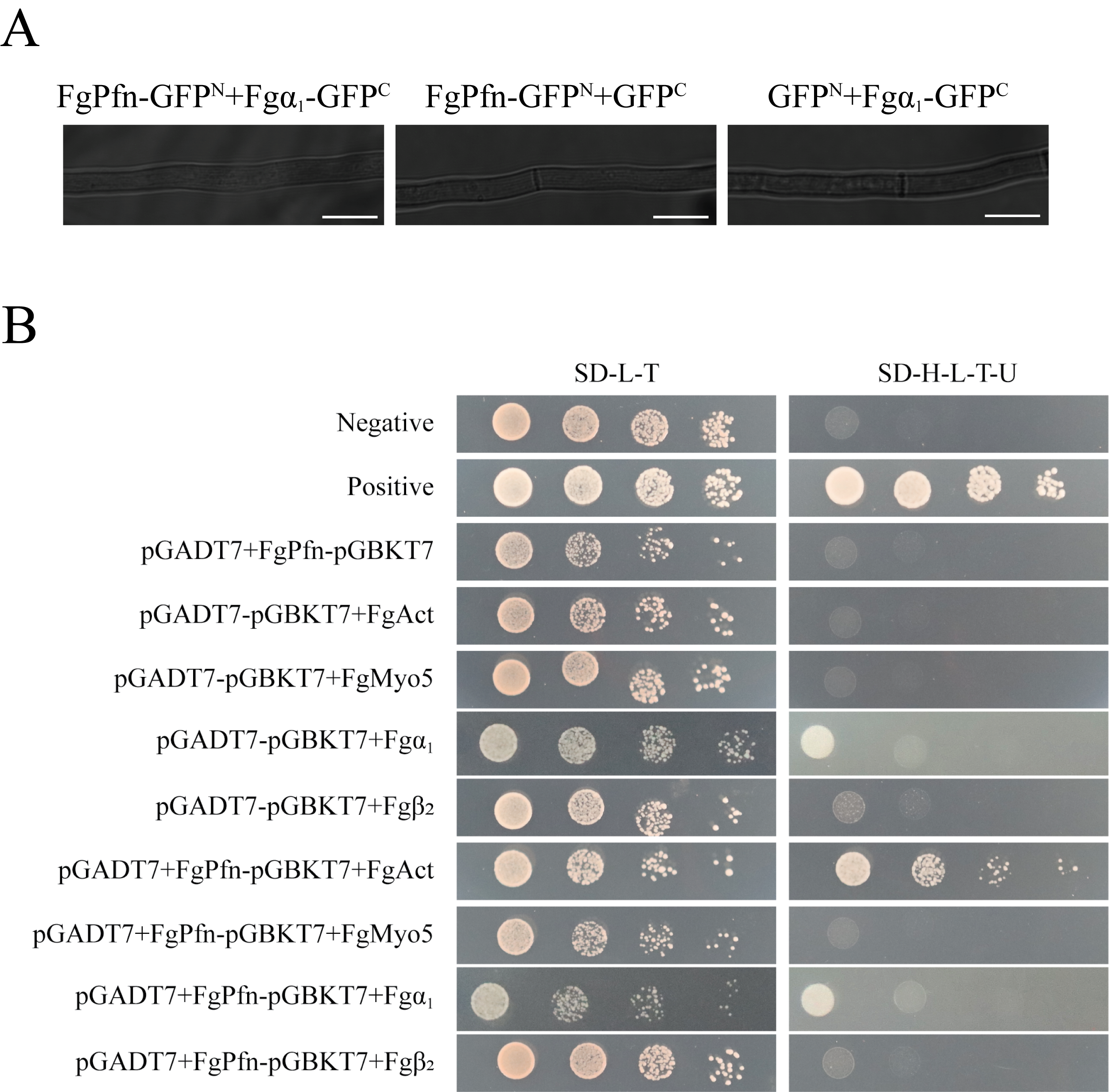

Supplement: S2 Fig — (A) Analysis of the interaction of FgPfn with Fgα1 by bimolecular fluorescence complementation (BiFC) assay. Bar = 10 μm.(B) Analysis of the interaction of FgPfn with FgAct, FgMyo5, Fgα1 and Fgβ2 by yeast two-hybrid (Y2H) assay. Saccharomyces cerevisiae AH109 strains containing the pGADT7 and pGBKT7 plasmid pairs can form colonies on SD-L-T medium, and the positive control and the interacting strains can form colonies on SD-H-L-T-U medium. (TIF) [file ppat.1012215.s002.tif]

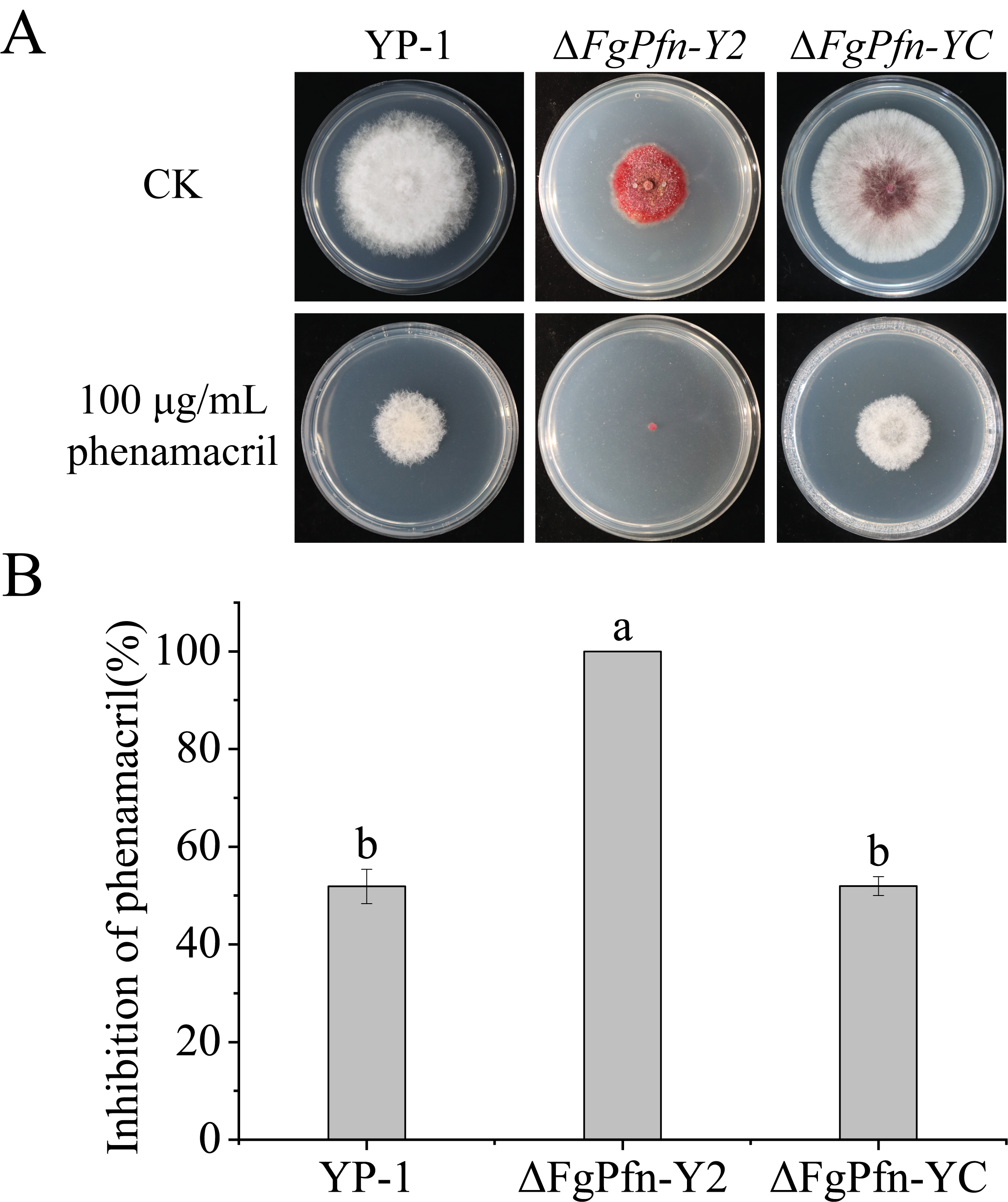

Supplement: S3 Fig — ΔFgPfn-Y2, from the highly phenamacril-resistant strain YP-1, also showed growing sensitivity to phenamacril compared to the parental strains and complementation strain. (TIF) [file ppat.1012215.s003.tif]

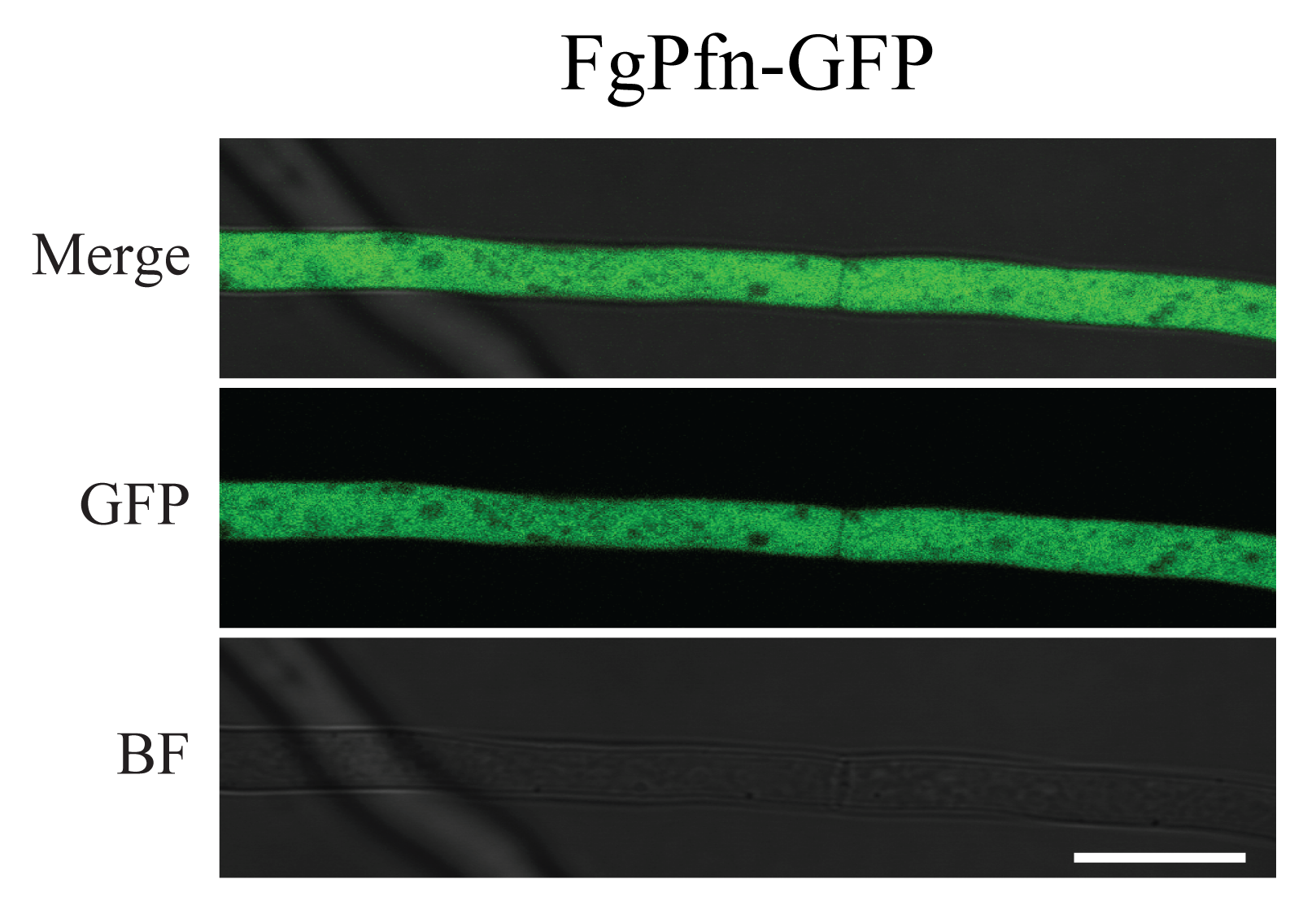

Supplement: S4 Fig — The strain was grown in YEPD for 36h and photographed under a confocal microscope. Bar = 10 μm. (TIF) [file ppat.1012215.s004.tif]

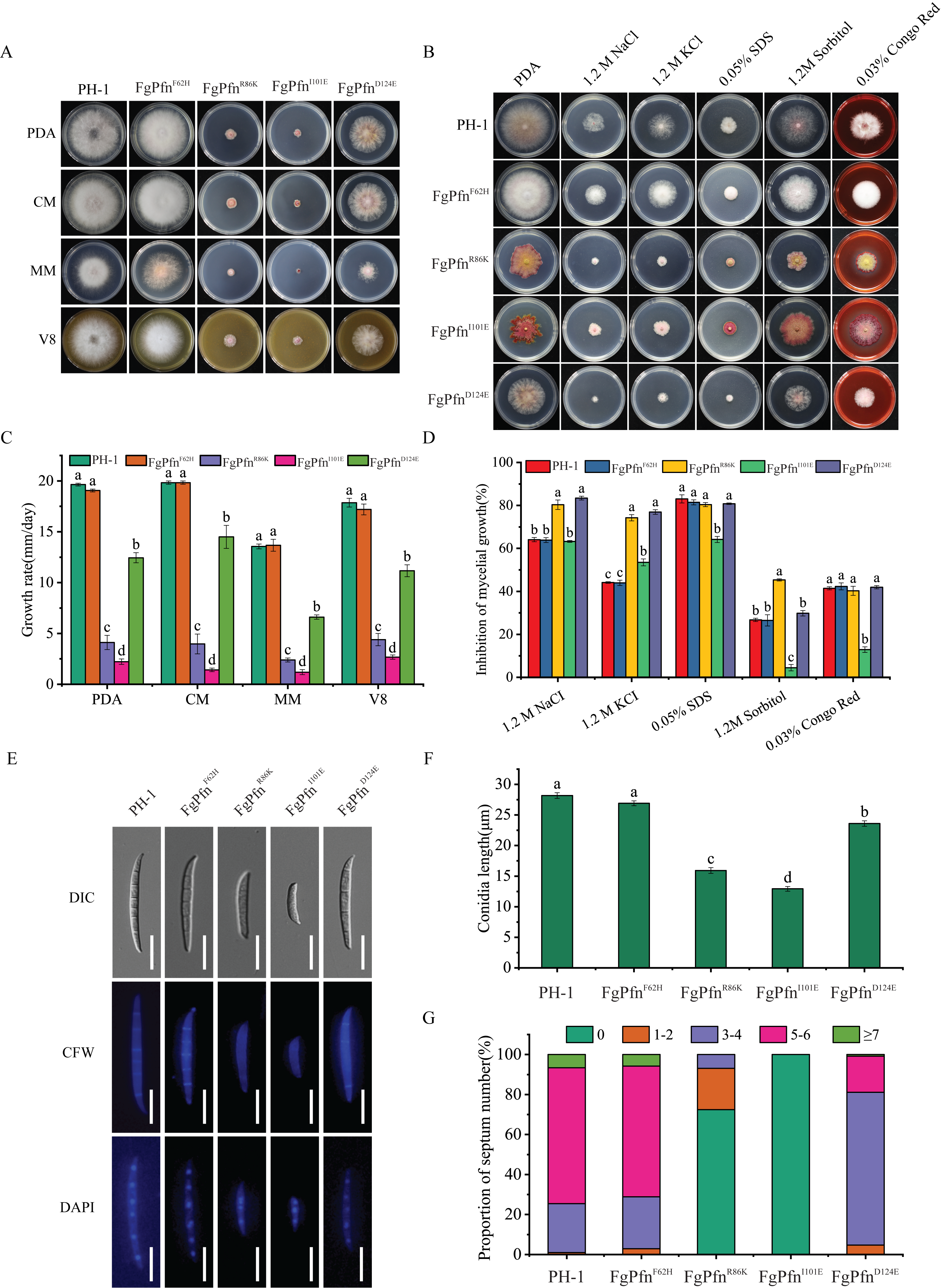

Supplement: S5 Fig — (A) Growth phenotype of different mutants. The colony morphology was photographed after the 3d inoculation of each strain on PDA, CM, MM, and V8 medium. (B) The colony morphology of different mutants on different medium. PH-1 and the mutation strains were inoculated on a PDA medium containing 1.2 M NaCl, 1.2 M KCl, 0.05% SDS, 1.2 M Sorbitol, and 0.03% Congo red at 25°C. The colony morphology of PH-1, F62H, and D124E strains was photographed after the 3d of growth, while R72E and R86K were photographed after 10d, and I101E was photographed after 20d of growth. (C) The mycelial growth rate of mutants was measured after the 3d growth on PDA, CM, MM, and V8 mediums. Bars with the same letter indicate no significant difference according to the least significant difference (LSD) test at p < 0.05. (D) The inhibitory effects of different stress factors on the hyphal growth of different strains were assessed by calculating the inhibition rates. The colony diameters of the strains grown on a PDA medium were used as the control. Inhibition rates = (Average diameters of control group—Average diameters of treatment group)/Average diameters of control group × 100%. Bars with the same letter indicate no significant difference according to the LSD test at p < 0.05. (E) Mutations of FgPfn resulted in conidial morphological defects. Conidia were examined by DIC microscopy. The septum of conidia was stained with CFW and photographed with an inverted fluorescent microscope at 20×. The nuclei of conidia were stained with DAPI for 30min and photographed with an inverted fluorescent microscope at 20×. Bar = 10 μm. (F) The average conidia length of each strain was measured with 100 conidia, which was repeated three times. Bars with the same letter indicate no significant difference according to the LSD test at p < 0.05. (G) The number of conidial septa of each strain was counted after staining with CFW, and then the proportion of conidia with different septate numbers to the total number w [file ppat.1012215.s005.tif]

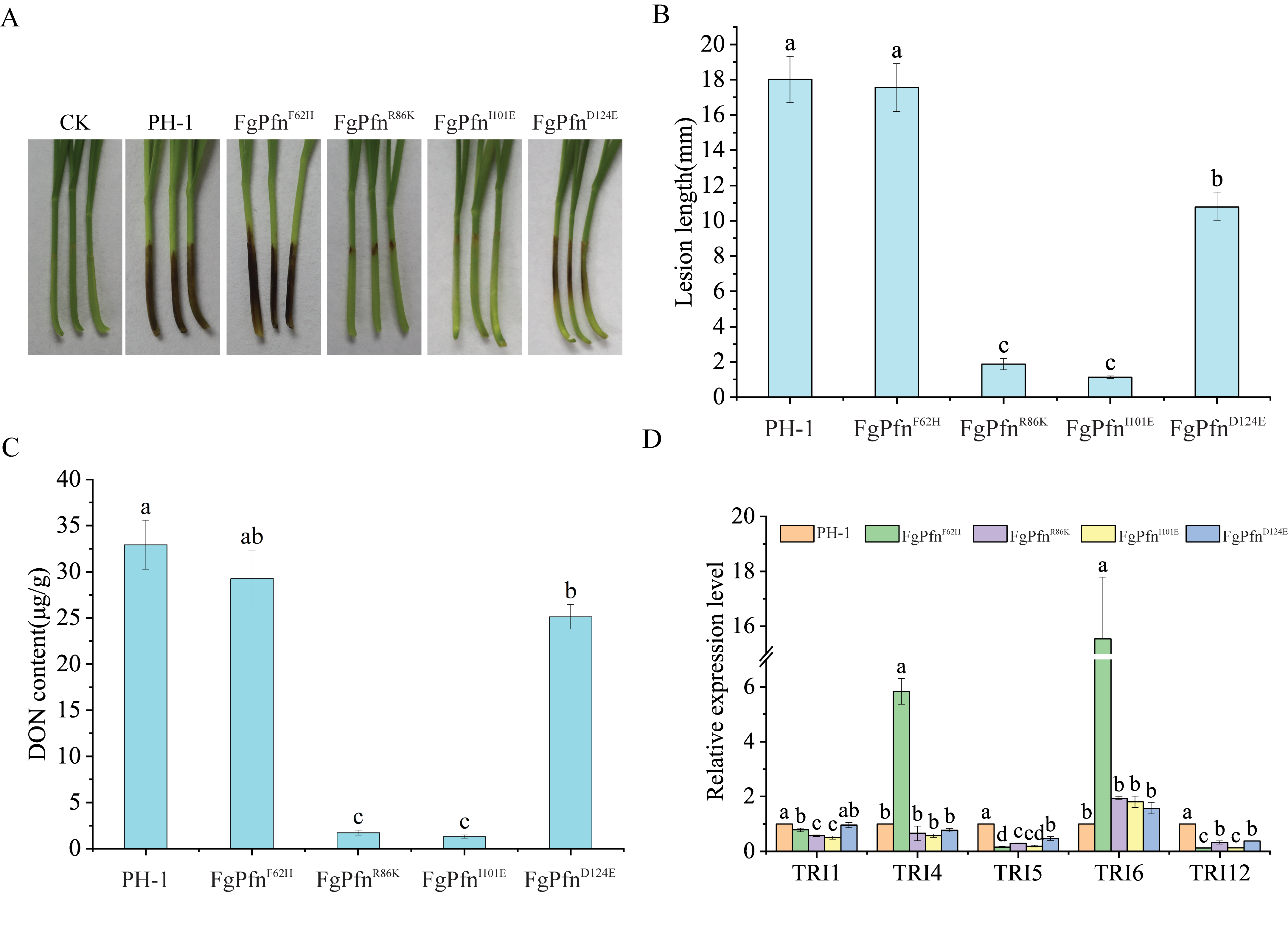

Supplement: S6 Fig — Conserved residue mutations in FgPfn affected pathogenicity (A) Pathogenicity assays of different mutants on wheat coleoptiles. Add 2 μL 1×10^6^ /mL conidia suspension was inoculated on the injured wheat coleoptile for 7d to observe the incidence and take photos. The wheat variety was Huaimai 33. (B) The average length of lesion on wheat coleoptile infected by each strain was measured after 7d post-inoculation. Bars with the same letter indicate no significant difference according to the LSD test at p < 0.05. (C) DON content assay of different mutants. After the 7d of TBI culture, the DON content in the wild-type PH-1 and mutation strains were determined. Bars with the same letter indicate no significant difference according to the LSD test at p < 0.05. (D) Relative gene expression level of TRI1, TRI4, TRI5, TRI6 and TRI12 in the strains tested. After the 36h culture in TBI, mycelia of each strain were harvested for RNA extraction. The GAPDH was used as a reference gene. Bars with the same letter indicate no significant difference according to the LSD test at p < 0.05. (TIF) [file ppat.1012215.s006.tif]
